# Correction: FisB relies on homo-oligomerization and lipid binding to catalyze membrane fission in bacteria

**DOI:** 10.1371/journal.pbio.3001499

**Published:** 2021-12-14

**Authors:** The PLOS Biology Staff

Supplemental Figures are mislabeled throughout the article as S1 Appendix A-L. Supplemental Figs should be labeled S1 Fig–S12 Fig in all article text and Supporting Information captions.

S1 Fig–S12 Fig are omitted from the list of Supporting Information. Please view S1 Fig–S12 Fig here.

S1 Appendix incorrectly contains captions for S1 Fig–S12 Fig. The correct version of S1 Appendix can be viewed below.

The captions for S1 Fig–S12 Fig are mislabeled. The correct captions for S1 Fig–S12 Fig can be viewed below.

The publishers apologize for these errors.

## Supporting information

S1 Appendix File containing Supporting experimental information, including quantification of FisB copy numbers using *B. subtilis* fluorescence standards and Western blotting, information showing that the localization of FisB is not coupled to cell wall remodeling, the proton motive force, or the membrane potential, data testing the topology of FisB, and Tables A–C. (DOCX)

S1 Fig Membrane fission assay. **A**. Membrane fission as a function of time after downshift to sporulation medium. Aliquots were taken from sporulating suspensions of wild-type (PY79) or FisB null mutant cells (BDR1083) at the indicated times, labeled with the membrane dye TMA-DPH, mounted on agarose pads containing resuspension medium, and imaged using a wide-field fluorescence microscope. The dye only partially crosses the membrane, resulting in strong labeling of the forespore contour pre-fission and dim labeling post-fission. **B.** The original membrane fission assay using the lipophilic membrane dye FM4-64 and expression of a forespore marker^17^. The dye is virtually non-fluorescent in the medium, and it cannot cross the cell membrane. Thus, before fission, FM4-64 labels the outer leaflet of both the mother cell and the forespore membranes. After fission, only the outer leaflet of the mother cell is labeled. Because post-fission cells and cells that never entered sporulation are labeled in the same manner, in addition to FM4-64, a fluorescent protein is expressed in the forespore under the control of the forespore-specific transcription factor σ^F^ to distinguish between the two cell types^6^. This makes it challenging to monitor FisB dynamics simultaneously, which requires a third channel. FM4-64 was added to an aliquot from a sporulating culture of *B*. *subtilis* cells (BKM15), then cells were mounted onto an agarose pad and imaged. The cell in the top image completed engulfment, but not fission, because the dye had access to the space around the forespore (FS). For the cells in the middle and bottom, the dye did not have access to the space around the FS. This could be because a cell never entered sporulation, or because of successful membrane fission. Expression of a FS reporter (PspoIIQ-cfp, green) is required to distinguish between the two possibilities. For the cell in the middle row, the FS marker is present, indicating the cell successfully underwent membrane fission. The cell in the bottom row never entered sporulation. **C**. Kinetics of membrane fission during sporulation, monitored using either FM4-64 or TMA-DPH. The two dyes result in indistinguishable kinetics. **D-G**. Quantification of TMA-DPH intensities for categorization of cells. Forespore contours were detected using JFilament^18^. A MATLAB script was then used to determine the mean fluorescence intensity along individual contours (see Materials and Methods). **D**. A snapshot of PY79 cells labeled with TMA-DPH at t = 2.5 h after downshifting into nutrient-poor medium. The same image is shown on the right, with detected forespore contours overlaid. **E.** Distribution of mean contour intensity values from the image in D. The distribution is bimodal. A fit to a sum of two Gaussian functions is shown, with means = 349, 459 a.u. and std. dev. = 35, 23, a.u. respectively. Cells having lower intensity values were classified as having undergone fission (red, 54/124 = 40%), while cells with higher intensity values (blue, 62/124 = 54%) were classified as "no fission". Eight cells (8/124 = 6%) displaying intermediate values were not classified. **F.** The same as in D, but cells were labeled at t = 3 h after nutrient downshift. **G.** Distribution of mean contour intensity values from the image in F. A double Gaussian fit yielded means = 333, 461 a.u. and std. dev. = 18, 22, a.u. respectively. Out of the 133 cells in F, 70% (93/133) were classified as having undergone fission, 23% (31/133) as "no fission", and 7% (9/133) as intermediate. For the data shown in Figs 1F, 3D, and 6D, the same analysis pipeline was used, but multiple view fields were analyzed from three independent experiments. Scale bars in A, D, F represent 1 μm.
(EPS)

S2 Fig Calibration of fluorescence intensity as a function of GFP copy number using *B*. *subtilis* calibration strains. **A.** Representative images of cells expressing known average copy numbers of sfGFP^1^. **B**. The total intensity (sum of pixel values) per cell, plotted against copy number, after selecting cell contours using MicrobeJ software and subtracting background. Background was defined as total intensity (sum of pixel values) in wildtype cells which did not express any fluorescent protein. Data is plotted on semi-logarithmic coordinates in the inset, to show low copy number intensities. **C**. *B*. *subtilis* cells expressing the same average number of mEGFP (BAL038) or sfGFP (SG13) were imaged to determine the correction factor for mEGFP-FisB copy number quantification. Top: representative images using the same acquisition and display settings, bottom: quantification. On a per molecule basis, sfGFP is ~2.4±0.2-fold brighter than mEGFP. Strains were made by transforming PY79 cells with either plasmid pECE321 (encoding amyE::(Pveg_R0_sfGFP_spec)) or its variant wherein sfGFP was replaced by mEGFP (pECE321_mEGFP). On average 3.00×10^5^ GFP copies/cell are expressed^1^. **D**. The calibration in B, rescaled with the correction factor determined in C, such that the calibration now corresponds to the copy numbers for mEGFP (the best-fit line constrained to pass through the origin has slope 22.48 (*R*^2^ = 0.93). **E.** Distribution of background-corrected total fluorescence intensity (sum of pixel values) for cells expressing mEGFP-FisB (BAL001) that have undergone membrane fission at t = 3h. Membrane fission was assessed as in Fig 1 and S1 Fig. Background was defined as in B. N = 250 cells. **F**. The total intensity of ISEP and DMCs as a percentage of total cell FisB fluorescence. Using the calibration for whole-cell fluorescence shown in D, ~1300 copies of FisB per cell; ~50 FisB/ISEP and ~16 FisB/DMC are estimated. Scale bars in A, C, represent 1 μm.
(EPS)

S3 Fig FisB copies per cell, estimated from Western blot analysis. **A.** Calibration of nanograms of YFP vs. WB band intensity. Top: purified mYFP was diluted in sporulation medium to indicated ng/lane, detected using chemiluminescence using an anti-GFP antibody (ab13970) and analyzed by densitometry. Bottom: amount of YFP (in ng) vs. integrated intensity of WB band (mean ± SEM of three independent experiments). Linear regression (dotted line, forced through the origin), with *y* = 2783*x* (*R*^2^ = 0.98). **B.** WB detection of mXFP-FisB (where X is Y or G) loaded from a known number of cells. Solubilized membranes of wild type (PY79), *∆fisB* (BDR1083), *∆fisB* expressing mYFP-FisB (BVS001) or mGFP-FisB (BAM003) at 3 h into sporulation, and standards of purified mYFP were separated by SDS-polyacrylamide gel electrophoresis, subjected to immunoblotting with an anti-GFP antibody and analyzed by densitometry. Each lane was loaded with 1.44×10^7^ cells, determined by serial dilutions and counting under the microscope. The average intensity of the mGFP-FisB and mYFP-FisB bands from the membrane fraction corresponds to 0.6±0.05 ng of mXFP-FisB in A, which in turn corresponds to (6.61±0.49)×10^9^ molecules per lane. Thus, on average there are 459±34 mXFP-FisB molecules/cell. However, some cleaved XFP (on average 44% of the mXFP-FisB membrane fraction signal) is detected in the soluble fraction, likely reflecting degradation of mXFP-FisB either in cells or during sample processing. Correcting for this, we estimate 661±49 mXFP-FisB molecules/cell.
(EPS)

S4 Fig FisB copy numbers in the low expression strain. **A.** Representative fluorescence images of cells expressing mEGFP-FisB under native (BAL001) or low (BAL004) expression levels. Cell contours (yellow outline) were calculated using MicrobeJ. Fission in each case was determined as in Fig 1C and S1 Fig. Due to the large difference in pixel values, images of native and low expression strains are displayed with different brightness settings. **B.** Comparison of total copies of FisB under native or low expression levels. Sum of pixel values (total mEGFP fluorescence) per cell was calculated with MicrobeJ within the cell contours as shown in A, after background correction. The calibration in Fig 2D was used to estimate copies per cell. Low-expression cells have 122±51 copies of mEGFP-FisB on average, or ~8-fold lower than native levels. **C.** Same as in A, with ISEP circled. ISEP were detected semi-automatically using SpeckleTrackerJ^19^. **D.** Distributions of total fluorescence intensities (sum of pixel values) for ISEP for the native and low expression strains. For each spot the sum of all pixel values in a 6×6 pixel (0.5 µm × 0.5 µm) box around the center of the cluster detected as in C was integrated. The same operation was performed at a membrane area where no clusters were present, and this background value was subtracted from the FisB cluster intensity. The integrated, background-corrected intensity values at the ISEP were compared directly with the calibration in Fig 2D. The distribution for native expression cells is copied from Fig 2C for comparison. Low expression cells have ~6 FisB at ISEP at the time of fission. **E.** The total intensity of ISEP as a percentage of total cell fluorescence for native and low expression cells. **F**. Immunoblot analysis of whole-cell lysates from sporulating cells. FisB levels were analyzed using an anti-FisB antibody in sporulating cells from wild-type (strain PY79), *ΔfisB* (BDR1083) and FisB-null cells expressing mYFP-FisB in low levels (strain BAL002). Time (in hours) after initiation of sporulation is indicated. **G.** Summary of FisB copy number quantification. Top. Under native expression, on average, there are ~1,000 FisB molecules per cell at t = 3 h. Each DMC contains ~12 FisB molecules, while the ISEP contains ~40 FisB copies. In the low-expression strain all the numbers are scaled down ~7-8-fold.
(EPS)

S5 Fig Sporulation efficiency of various *B*. *subtilis* strains. Sporulation efficiency was assayed by measuring heat-resistant (80°C, 20 min) colony forming units (see Materials and Methods) and normalized to the wild-type level (% of WT) for the indicated strains. Results are shown as means ± SD for four replicates per condition.
(EPS)

S6 FigMotion of FisB clusters is not coupled to cell wall synthesis, or pH and voltage gradients across the cell membrane.**A.** Representative TIRFM images of cells expressing GFP-Mbl (BDR2061) before and after treatment with fosfomycin. Red and light blue lines indicate the directions along the long and short axes of the cell used to compute the kymographs on the right. Before treatment, GFP-Mbl moved around the cell circumference, reflected by stripes across the cell in the maximum intensity projections (MIP) and spots that appear and disappear in the kymographs along the long axis (marked with a red frame). Spots also appear and disappear along the short axis as GFP-Mbl spots move in and out of the evanescent field as they move along the cell circumference. Addition of fosfomycin stopped the motion of GFP-Mbl, reflected in small spots in the MIP and continuous lines in the kymographs. **B**. Mean-squared displacement (MSD) as a function of lag time for GFP-Mbl before (24 tracks) and after (20 tracks) fosfomycin treatment. Colored lines connect averaged points, whereas gray areas represent standard deviation and error bars represent the standard error of the mean (SEM). Movies were acquired at 1 frame/s. The short-time diffusion coefficient, estimated from a parabolic fit to the MSD, was *D*_*Mbl*_ = 505 *nm*^2^/*s* (95% confidence interval CI = 439–571 *nm*^2^/*s*) and $D_{Mbl}^{fos}=112\;nm^{2}/s$ (CI = 79–146 *nm*^2^/*s*) before and after fosfomycin treatment, respectively. **C**. Representative TIRFM images of cells expressing mGFP-FisB (BMB014). Motion of GFP-FisB was not affected by addition of fosfomycin. **D**. MSD as a function of lag time for GFP-FisB before (18 tracks) and after (12 tracks) fosfomycin treatment. Acquisition rate was 1 frame/s. The short-time diffusion coefficient was *D*_*FisB*_ = 6270 *nm*^2^/*s* (95% confidence interval CI = 5810–6740 *nm*^2^/s) and $D_{FisB}^{fos}=6370\;nm^{2}/s$ (CI = 5580–7160 *nm*^2^/*s*) before and after fosfomycin treatment, respectively. **E**. Average total distance traveled by GFP-Mbl and mGFP-FisB spots over 3 s in the presence and absence of fosfomycin. GFP-Mbl (20 tracks), GFP-Mbl + fosfomycin (24 tracks), mGFP-FisB(18 tracks) and mGFP-FisB + fosfomycin (12 tracks). Fosfomycin decreased the total distance traveled by Mbl filaments (*p* = 0.024, Student’s t-test), whereas FisB was not affected (*p* = 0.433). **F**. Average asymmetry of the Mbl and FisB trajectories. Upon treatment with fosfomycin, GFP-Mbl filaments stop moving, which is reflected as a decrease in asymmetry (*p* = 0.0044), whereas mGFP-FisB’s motion is unaffected (*p* = 0.8655). **G.** Localization of GFP-Mbl (BDR2061) during vegetative growth and mGFP-FisB (BAM003) at t = 3h into sporulation in the presence or absence of 100 μM CCCP or 30 μM valinomycin. GFP-Mbl mislocalizes in the presence of either drug, whereas the localization of mGFP-FisB is unaffected. Scale bar is 3 μm.(EPS)

S7 Fig Conservation and predicted topology of FisB. **A**. Conservation of FisB amino acid sequences derived from alignment of 250 FisB sequences from the SwissProt database, using the program ConSurf version 3.0^20^. **B.** Membrane protein topology prediction from 10 different algorithms, and the consensus prediction by Constrained Consensus Topology Prediction Server (CCTOP^21^).
(EPS)

S8 Fig Domain structure and topology of *B*. *subtilis* FisB. **A.** Predicted domain structure of FisB. Pfam^14^ identifies a consensus region (residues 129–223) defining the FisB protein family. **B**. Kyte-Doolittle hydrophobicity profile of the FisB sequence, with a potential second TMD indicated **C**. Possible topologies of FisB. Left: a single TMD with a cytoplasmic N-terminus and extracellular C-terminus. Right: With two TMDs, both the N- and the C-termini should be cytoplasmic. Cysteine residues introduced at positions 6 or 245 are indicated. **D**. Accessibility of the cysteines at positions 6, 137, and 245 to a biotinylated, sulfhydryl-reactive compound, 3-(N-maleimidoypropionlyl) biocytin (MPB). Myc-tagged monocysteine FisB variants were produced in *ΔfisB* cells and reacted with MPB before or after blocking extracellular cysteines with 4-acetamido-4’-maleimidylstilbene-2,2’-disulfonic acid (AMS). FisB was pulled down using an anti-myc antibody and biotinylation was probed by Western blot using an HRP-conjugated avidin antibody. Lysed cells were probed to ensure accessibility of MPB to the cysteine labels. The results are consistent with the amino and carboxy termini being intra- and extracellular, respectively.
(EPS)

S9 Fig His6-FisB ECD forms soluble aggregates *in vitro* and binds acidic membranes mainly through electrostatic interactions. **A.** Schematic domain structure of the 6His-tagged FisB construct comprising the soluble extracytoplasmic domain (ECD), generated for recombinant protein purification. **B.** Second and third elution fractions from the affinity column were analyzed by SDS-PAGE and stained with SpyroOrange. The red arrow indicates the monomeric form of His6-FisB (23 kDa) and the blue bracket highlights SDS-resistant His_6_-FisB multimers. **C.** Gel filtration elution profile of His6-FisB^ECD^ in Superose 6 Increase 10/300 GL column (top). Two fractions comprising the indicated peaks were re-injected in the same column under the same conditions, and eluted at the same volume as in the original sample. Elution volumes of molecular weight markers are indicated. **D.** Peaks labeled 1–3 in C were analyzed by Coomassie Blue-stained SDS-PAGE. Molecular weight markers are indicated on the left (in kDa). The band that corresponds to His_6_-FisB^ECD^ is indicated with a red arrow. The black asterisk indicates a chaperone that co-elutes with monomeric His_6_-FisB^ECD^. **E.** Representative electron micrographs of fractions comprising the 1st and 2nd peaks from C. Scale bar is 50nm. **F-H.** FisB ECD binding to liposomes is independent of calcium or pH, but decreases rapidly with increasing ionic strength. SUVs composed of 45 mole % CL and 55 mole % PC (40 nmol total lipid) were incubated with 200 pmol His_6_-FisB ECD in buffers with the indicated [Ca^2+^] (F), pH (G), or NaCl (H) for 1h and subjected to step-gradient isopycnic ultracentrifugation.
(EPS)

S10 Fig FisB mutants selectively deficient in membrane binding or oligomerization are expressed at similar levels as wild-type FisB. **A.** Examples of cell contours detected using MicrobeJ. **B**. Distributions of background-corrected total fluorescence intensity per cell for *ΔfisB* cells expressing mYFP-FisB^WT^ (BAL002), mYFP-FisB^KK^ (BAL006), or mYFP-FisB^GIII^ (BAL007) at low levels. The pixel values within the contours detected by MicrobeJ as in A were summed to define the total intensity per cell. This value was corrected for autofluorescence and background by subtracting the average total intensity per cell in cells (PY79) that did not express any fluorescent protein. The three distributions were indistinguishable, indicating that the mutants were expresses at the same level as the wild-type protein. **C**. Expression levels of mYFP-FisB^GIII^ (BAL007) was similar to those of FisB^WT^ (BAL002) using Western blotting, probed using an anti-FisB antibody. Time points into sporulation probed are indicated above the blot.
(EPS)

S11 Fig FisB mutants tested. **A.** Mutations neutralizing 1–4 positively charged residues in the consensus region were introduced into FisB ECD, the mutants were expressed in *E*. *coli*, purified, and tested for binding to negatively charged liposomes using the flotation assay depicted in Fig 4C. Neutralization of lysines around K170 produced the strongest reduction in binding. Liposomes were composed of 45 mole % CL and 55mole % PC**. B**. Other designed mutations targeted hydrophobic residues (black), inversion of positively (blue) or negatively (red) charged residues, or deletions. mYFP fusions of the mutated FisB were expressed at low levels in *ΔfisB* cells and tested for heat-resistant colony formation (C,D) and imaged for localization (E). **C.** Sporulation efficiency of cells expressing mYFP-FisB with deletion and hydrophobic residue mutations shown in B. **D**. Sporulation efficiency of cells expressing mYFP-FisB with charge inversion mutations shown in B. **E**. Images of sporulating cells (t = 3 h) expressing mYFP-FisB bearing some of the mutations in C,D. In half the cases, the mYFP signal was cytosolic, suggesting the fusion protein was not inserted into the membrane and degraded (images boxed in red). In other cases, some mYFP signal was on the membrane and some was cytosolic (cyan-framed images). Cases in which mutants were located exclusively to the membrane were rare and included neutral mutations (images boxed in green) as well as FisB^KK^ and FisB^GIII^ shown in Fig 6, at least under low expression levels. Scale bar represents 1 μm.
(EPS)

S12 Fig Alignment of B. subtilis and C. perfringens FisB sequences. *B*. *subtilis* (uniprot ID O32131, *YUNB_BACSU*) and *C*. *perfringens* (uniprot ID A0A0H2YVA3, *A0A0H2YVA3_CLOP1*) sequences were obtained from The Universal Protein Resource (UniProt) database (www.uniprot.org) and aligned using Clustal Omega^22^ (https://www.ebi.ac.uk/Tools/msa/clustalo/).
(EPS)
