# Supplementary material for: FisB relies on homo-oligomerization and lipid binding to catalyze membrane fission in bacteria
Source: PLoS Biol. 2021 Jun 29;19(6):e3001314. doi: 10.1371/journal.pbio.3001314 (PMC8274934; doi:10.1371/journal.pbio.3001314)
Supplement: S2 Appendix — (PDF) [file pbio.3001314.s002.pdf]

S2 Appendix: Theoretical Modeling

DERIVATION OF THE EQUILIBRIUM EQUATIONS

As explained in the main text, we consider the free energy $\mathscr{F}$ of an axisymmetric cylindrical membrane neck of radius R and length L that connects two planar membrane sheets, corresponding to the local geometry depicted in **Fig A**, where the engulfment membrane meets the rest of the mother cell membrane. For simplicity, we assume the surface density ϕ of FisB proteins in the neck is uniform and reaches equilibrium with a surface density ϕ_0 of FisB in the surrounding planar membranes. The energy functional consists of a term accounting for membrane bending and tension, $\mathscr{F}_{\mathrm{m}}$, and another term accounting for FisB protein-protein interactions, $\mathscr{F}_{\mathrm{p}}$. We employ the classical Helfrich-Canham theory [1–6] for the energy of the membrane, $\mathscr{F}_{\mathrm{m}}$, which reads

$$\mathscr{F}_{\mathrm{m}} = \int_{S_{\mathrm{n}}} \mathrm{d}S_{\mathrm{n}} \left[\frac{\kappa}{2}H^2 + \gamma\right] + \int_{S_{\mathrm{s}}} \mathrm{d}S_{\mathrm{s}}\gamma, \tag{1}$$

where S_{n} and S_{s} are the surfaces of the membrane neck and sheets, H is twice the mean curvature, κ is the bending modulus, and γ is the surface tension. The two surrounding membrane sheets are assumed to be planar since their curvature is much less than that of the neck, thus their only contribution to the energy comes from membrane tension.

Concerning FisB proteins in the neck, we include translational entropy, the energy of homo-oligomerization in trans crossing the interior of the neck, and an energy that limits crowding. Additionally, as shown in the main text, FisB proteins do not exhibit curvature sensing, thus we do not include a term coupling FisB density to spontaneous curvature in Eq. (1). This results in the following expression for the protein free energy functional [7–10]

$$\mathscr{F}_{\mathrm{p}} = \int_{S_{\mathrm{n}}} \mathrm{d}S_{\mathrm{n}} \left\{k_{\mathrm{B}}T\phi \ln\left(\frac{\phi}{\phi_0}\right) + aV_{\mathrm{LJ}}(R)\phi^2 + U(\phi)\right\}. \tag{2}$$

The first term accounts for translational entropy. The second term is an energy per unit area describing trans interactions of FisB, where a is the energy per FisB density, and which for simplicity is assumed to be proportional to the standard Lennard–Jones (LJ) potential accounting for a longer-range attraction and shorter-range repulsion:

$$V_{\mathrm{LJ}}(r) = \left(\frac{\sigma}{r}\right)^{12} - \left(\frac{\sigma}{r}\right)^{6}, \tag{3}$$

σ being the length scale at which the energy crosses from repulsive to attractive. The function $U(\phi)$ is an energy penalty for in-plane crowding that increases rapidly above a certain FisB concentration. To obtain $U(\phi)$ we assume a purely repulsive, truncated and shifted LJ potential between cis-neighboring FisB molecules, which we take to occupy a triangular lattice in order to relate density to nearest-neighbor distance. Therefore, $U(\phi) = \varepsilon[V_{\mathrm{LJ}}(r(\phi)) - V_{\mathrm{LJ}}(r_{\max})]$ when

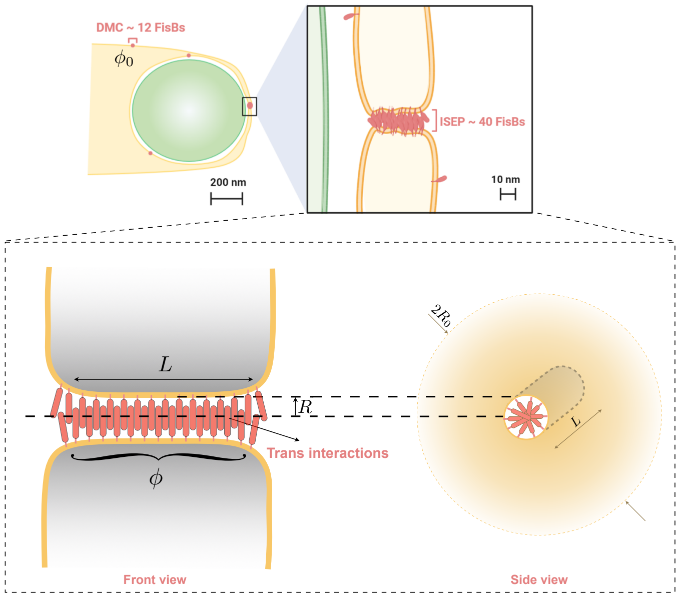

Fig A. Left: Sketch of the late stages of forespore engulfment, when a small membrane neck connects the engulfment membrane to the rest of the mother cell membrane. Right: Schematic of FisB accumulation at the fission site. FisB freely moves around the engulfment membrane and other regions of the mother cell membrane, forming clusters of up to $\sim$12 molecules. Bottom: Sketch of the modeled axisymmetric membrane neck of radius R and length L populated by a uniform surface density ϕ of FisB, and which connects two planar membrane sheets. The side view of the neck displays the idealized distance R_0 at which FisB surface density becomes ϕ_0. (Reproduced from Panel A of Fig 9 of the main text.)

$r \le r_{\max}$ and 0 when $r > r_{\max}$, where we have chosen $r_{\max} = 2^{1/6}\sigma_{\mathrm{cis}}$, namely the minimum of the LJ potential with length scale σ_{cis}. The result is

$$U(\phi) = \begin{cases} \varepsilon \dfrac{(\phi^3 - \phi_{r_{\max}}^3)^2}{4\phi_{r_{\max}}^6} & \phi \ge \phi_{r_{\max}}, \\ 0 & \phi < \phi_{r_{\max}}, \end{cases} \tag{4}$$

where $\phi_{r_{\max}} = 2^{2/3}/(3^{3/2}\sigma_{\mathrm{cis}}^2) = 2/(3^{3/2}r_{\max}^2)$ is the FisB concentration corresponding to a nearest neighbor distance $r_{\max}$.

To obtain an equation for the equilibrium density of FisB proteins in the neck, we minimize $\mathscr{F} = \mathscr{F}_{\mathrm{m}} + \mathscr{F}_{\mathrm{p}}$ with respect to ϕ,

$$k_{\mathrm{B}}T[1 + \ln(\phi/\phi_0)] + 2a\phi\left[\left(\frac{\sigma}{R}\right)^{12} - \left(\frac{\sigma}{R}\right)^{6}\right] + \partial_\phi U(\phi) = 0. \tag{5}$$

Then, minimizing $\mathscr{F}$ with respect to R yields an equation that determines the equilibrium radius of the neck

$$\gamma_{\mathrm{eff}} - \frac{\kappa}{2R^2} + a\phi^2\left(\frac{6\sigma^6}{R^6} - \frac{12\sigma^{12}}{R^{12}}\right) - \frac{2\gamma R}{L} = 0, \tag{6}$$

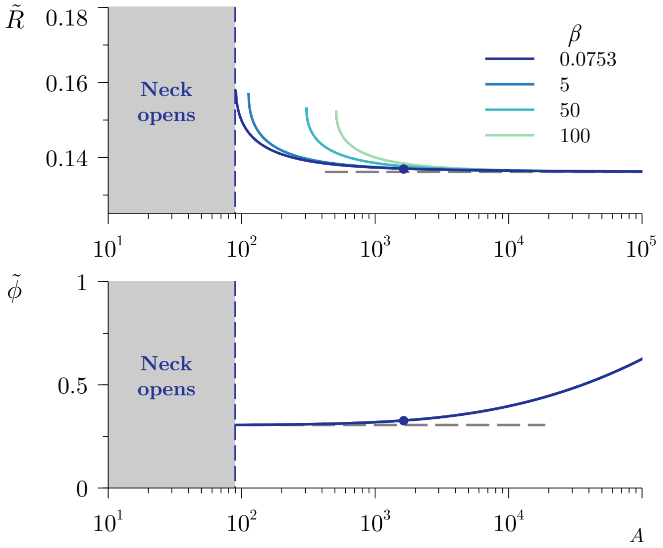

Fig B. Dimensionless equilibrium neck radius R (top) and FisB surface density ϕ (bottom) as functions of the dimensionless FisB trans homo-oligomerization strength A, for $\Gamma = 0.149$, $\Lambda = 6.11\times10^{-4}$, $\bar{\varepsilon} = 200$, $\Sigma = 0.121$, and different values of β indicated in the legend. Below a minimum interaction strength, FisB cannot stabilize the neck and the neck opens. The horizontal lines are the radius corresponding to the minimum of the potential describing the trans interaction, $R = 2^{1/6}\Sigma$, and the concentration of FisB at the onset of in-plane crowding $\tilde{\phi}_{r_{\max}} = 2^{2/3}/3^{3/2}$. The dot indicates the predicted equilibrium neck radius and the corresponding FisB surface density in the neck for the values of the dimensionless parameters obtained using the estimates given in **Table A**, namely $A \simeq 1.637\times10^3$ and $\beta \simeq 0.075$.

Table A. Estimates of the physical parameters

Parameter	Value
κ	$20 k_B T$ [11]
ϕ_0	100 FisB μm^{-2}
γ	10^{-4} N m^{-1} [12]
$\phi_{r_{\max}}$	5×10^4 FisB μm^{-2}
a	$10^4\ k_B T$ nm^2 FisB^{-1}
L	40 nm
$\sigma\sim\sigma_{\mathrm{cis}}$	2.47 nm
ε	32.78 $k_B T$ nm^{-2}
R_0	1.5 μm
D	0.1-1 μm^2/s

where $\gamma_{\mathrm{eff}} = \gamma + k_B T\phi\ln(\phi/\phi_0) + U(\phi)$.

At this point, it is important to emphasize that thermal fluctuations as well as hydrodynamics, which are neglected here, may play an important role during membrane fission. However, our aim here was specifically to model the formation of a stable FisB cluster in the neck prior to fission. To this end, we took into account only the essential protein-protein interactions and a simplified version of the geometry within a mean field model.

DIMENSIONLESS PARAMETERS AND EQUATIONS

To reduce the parameters of the system, Eqs. (5) and (6) are non-dimensionalized by choosing $\phi_c = \sigma_{\mathrm{cis}}^{-2}$ and $R_c = \sqrt{\kappa/(2\gamma)}$ as the characteristic scales of FisB surface density at the neck, and the neck radius, respectively. In particular, using the estimates in **Table A**, $\phi_c \simeq 1.637\times10^5$ FisB μm^{-2} and $R_c \simeq 20.28$ nm. The value of σ_{cis} is obtained from the relation $\phi_{r_{\max}} = 2^{2/3}/(3^{3/2}\sigma_{\mathrm{cis}}^2) = 2/(3^{3/2}r_{\max}^2)$. Introducing these scales into Eqs. (5) and (6) yields

$$1+\ln\left(\frac{\tilde{\phi}}{\Lambda}\right) + 2A\tilde{\phi}\left[\left(\frac{\Sigma}{\tilde{R}}\right)^{12} - \left(\frac{\Sigma}{\tilde{R}}\right)^{6}\right] + \partial_{\tilde{\phi}}\tilde{U}(\tilde{\phi}) = 0, \tag{7a}$$

$$\Gamma\left(1-\frac{1}{\tilde{R}^2}\right) + \tilde{\phi}\ln\left(\frac{\tilde{\phi}}{\Lambda}\right) + \tilde{U}(\tilde{\phi}) + A\tilde{\phi}^2\left[6\left(\frac{\Sigma}{\tilde{R}}\right)^{6} - 12\left(\frac{\Sigma}{\tilde{R}}\right)^{12}\right] - 2\beta\tilde{R} = 0, \tag{7b}$$

where

$$\tilde{U}(\tilde{\phi}) = \begin{cases} \dfrac{\tilde{\varepsilon}}{192}(4\sqrt{3} - 243\tilde{\phi}^3)^2 & \tilde{\phi} \geq \dfrac{2^{2/3}}{3^{3/2}}, \\ 0 & \tilde{\phi} < \dfrac{2^{2/3}}{3^{3/2}}, \end{cases} \tag{8}$$

where $\tilde{\phi}$ and $\tilde{R}$ denote the dimensionless versions of FisB surface density and neck radius, respectively. The dimensionless parameters in Eqs. (7a) and (7b) are

$$A = \frac{a\phi_c}{k_B T}, \quad \Sigma = \frac{\sigma}{\sqrt{\kappa/(2\gamma)}}, \quad \tilde{\varepsilon} = \frac{\varepsilon\sigma_{\mathrm{cis}}^2}{k_B T}, \quad \Lambda = \frac{\phi_0}{\phi_c}, \quad \Gamma = \frac{\gamma}{k_B T\phi_c}, \quad \beta = \frac{\gamma\sqrt{\kappa/(2\gamma)}}{k_B T\phi_c L}. \tag{9}$$

Taking the values from **Table A**, $\Gamma \simeq 0.149$, $\Lambda \simeq 6.11\times10^{-4}$, $\beta \simeq 0.075$, $A \simeq 1.637\times10^3$, $\tilde{\varepsilon} \simeq 200$, and $\Sigma \simeq 0.121$.

It is important to point out that the equilibrium radius in the presence of FisB proteins is significantly smaller than the expected length of the neck. Hence, in this configuration, we expect that boundaries do not play a significant role in the minimization problem described above, since $L \gg R$, and thus $\Delta L \sim R \ll L$, where ΔL is the characteristic length of the boundaries. Indeed, the dimensionless parameter that characterizes the importance of boundary effects is β, which is small according to the estimates of Table A, namely $\beta \sim 10^{-1}$, as given above. Therefore, we have neglected the small contribution from the boundaries in solving the minimization problem.

CONDITIONS FOR A STABLE NECK RADIUS IN THE PRESENCE AND IN THE ABSENCE OF FisB

Fig B shows $\tilde{R}$ and $\tilde{\phi}$ as functions of the dimensionless FisB trans homo-oligomerization strength A, for different values of

β indicated in the legend. In particular, fixing the material parameters given in **Table A**, β is an inversely-proportional function of the neck length L alone. Indeed, for a realistic estimate of the material parameter values (the dot), we find that FisB trans interactions are strong enough to stabilize the neck at $\tilde{R} \sim 0.14$, i.e. $R = 2.84$ nm, with a close-packed concentration of FisB in the neck $\tilde{\phi} \sim 2^{2/3}/3^{3/2}$, i.e. $\phi = 0.05$ FisB nm^{-2}. The predicted equilibrium radius is quite small, being close to the limit where the applicability of the continuum Helfrich model may be questioned. Nonetheless, there are several works where the Helfrich model has been employed satisfactorily at these small scales [10, 13].

We also show that there is a critical lower limit of A below which the FisB trans interactions are too weak to stabilize the neck, thus the neck opens ($\tilde{R} \to \infty$) in our simplified model. This critical value of A depends on β, namely the larger the value of β (the shorter the neck), the stronger the trans interactions needed to stabilize the neck at a finite radius. This makes intuitive sense: the longer the neck, the more FisB can be present to hold the neck together in opposition to membrane tension, which tends to make the neck expand.

We note that even in the absence of FisB, for a long enough neck there is a metastable state of the neck at finite radius R [14, 15]. This reflects a balance between membrane bending energy, which always favors larger R, and surface tension, which contributes non-mononotically to the membrane energy as a function of R. For small R and large L, increasing the radius R increases the total amount of membrane in the vicinity of the neck and so increasing R is opposed by surface tension. By contrast, for large R, increasing R removes more membrane from the parallel sheets than is added to the neck, since the former scales $\sim R^2$ while the latter scales $\sim LR$, so surface tension favors further increase of R. To illustrate this effect mathematically we analyze the membrane free energy functional in the absence of FisB proteins, which reads

$$\mathscr{F}_{\mathrm{m}} = R - \frac{R^2}{L/R_{\mathrm{c}}} + \frac{1}{R}. \quad (10)$$

For lengths below a critical value $L < 3^{3/2}R_{\mathrm{c}}$, the above energy functional does not have a real minimum and expanding the radius of the neck decreases the total energy of the system, implying the neck opens. By contrast, for $L > 3^{3/2}R_{\mathrm{c}}$, $\mathscr{F}_{\mathrm{m}}$ in Eq. (10) has a local minimum, implying a metastable finite neck radius. For the sake of conciseness the lengthy expression for the metastable radius is not shown here.

MINIMUM STABLE NECK LENGTH

In this section we compute the critical β (i.e. neck length) at which the stable neck represented by the dot in **Fig B** opens for realistic parameter values. For a value of $A \simeq 1.637 \times 10^3$, the opening of the neck occurs at $\beta \simeq 402$, which, taking the estimates from **Table A**, corresponds to a value of L below 1 Å. This means that for realistic values of L and the estimates given in **Table A**, our simple model always predicts the membrane neck can be stabilized by FisB trans interactions.

DIFFUSION AND CAPTURE TIME ESTIMATE

While the above results suggest that an accumulation of FisB at the neck can be energetically stable, we now consider how long it might take to reach that state. Here, we first analyze the time needed for FisB monomers in the surrounding membranes to diffuse and be captured at the neck.

To estimate the time required for a certain number of FisB proteins to be absorbed by the neck, we consider purely-diffusive dynamics within a planar membrane connected to the neck. In particular, we assume an annular domain, $\mathscr{V}$, depicted in **Fig A** (bottom right), whose FisB concentration $\Phi_0(\boldsymbol{x}, t)$ is given by the diffusion equation, which is made dimensionless employing

$$\phi_{0,\mathrm{c}} = \phi_0, \quad \ell = R_0, \quad t_{\mathrm{c}} = \frac{R_0^2}{D} \quad (11)$$

as characteristic scales for the areal FisB concentration, length, and time, respectively. Here ϕ_0 is the concentration of FisB in the sheet, assumed to be held constant at a certain radius R_0 far from the neck, and D is the diffusion coefficient of FisB. The dimensionless diffusion equation then reads

$$\partial_t \Phi_0 = \boldsymbol{\nabla}^2 \Phi_0 \quad \boldsymbol{x} \in \mathscr{V}, \quad (12)$$

with $\boldsymbol{x} = (r, \theta)$ denoting the position vector. We impose an absorbing boundary condition at the neck radius R, and a fixed value of Φ_0 at the outer radius R_0,

$$\Phi_0 = 0 \quad \text{at } r = \eta, \quad \text{and} \quad \Phi_0 = 1 \quad \text{at } r = 1, \quad (13)$$

where $\eta = R/R_0$ is the inner-to-outer radii ratio. Concerning the initial condition, as a conservative choice we impose $\Phi_0(t = 0) = 0$ everywhere except at the outer radius, where $\Phi_0(t = 0, r = 1) = 1$. The solutions can be found using, for example, a Fourier expansion involving Bessel functions (not shown here for the sake of brevity). The axisymmetric time-dependent solution is shown in **Fig C** at different times indicated in the legend. The inset displays the flux at $r = \eta$ as a function of time, from which we can obtain an estimate of the absorption time of a specified number of FisB proteins at the neck. At steady state, the concentration of FisB proteins in the annulus and the corresponding flux at the neck, $r = \eta$, read

$$\Phi_0(r, t \gg 1) = 1 - \frac{\ln(r)}{\ln(\eta)}, \quad f(t \gg 1) = -\frac{2\pi}{\ln(\eta)}. \quad (14)$$

Using the estimates given in **Table A**, where we have considered $R_0 \simeq 1.5$ μm, the characteristic diffusion time is $t_{\mathrm{c}} = R_0^2/D \simeq 2.25$-$22.5$ s, and the ratio of radii is $\eta \simeq 4 \times 10^{-3}$. The steady-state flux then yields, $f \simeq 1.126$, i.e. 10 FisB s^{-1}. Using the time-dependent evolution of the flux, the time required for diffusion and capture ~40 FisB proteins in the neck is ~4 s, which is very short and thus not expected to be rate limiting.

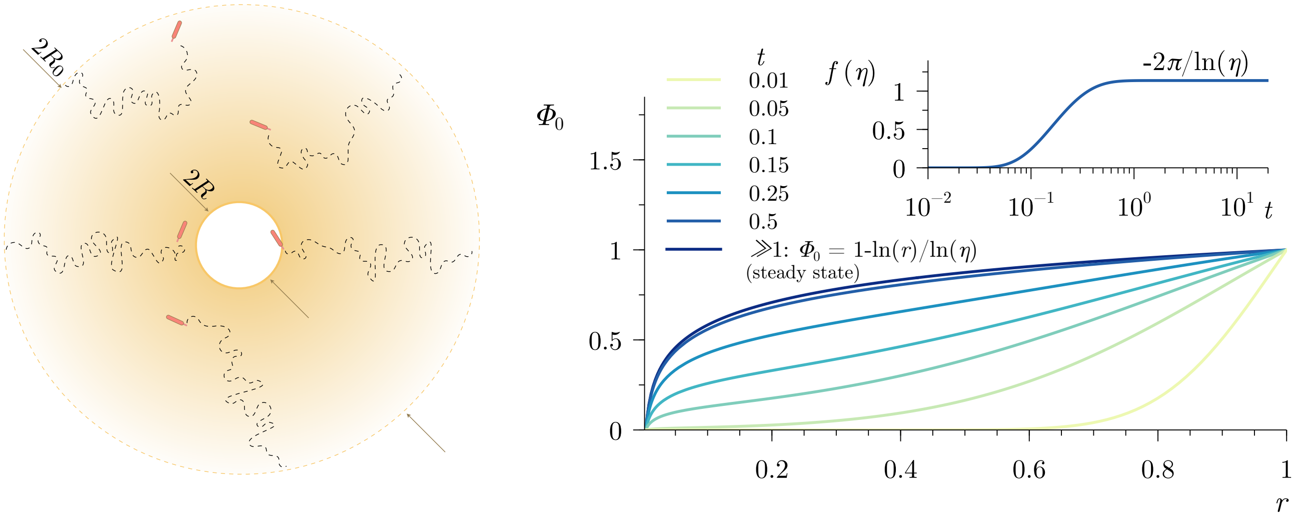

Fig C. Left: Sketch of the diffusion and capture process of FisB monomers. To estimate the minimum time required for FisB to accumulate in the neck, monomers are assumed to diffuse in the surrounding planar membranes and to be captured when they reach the neck. Right: Concentration of FisB, $\Phi_0(r)$ at the membrane sheet as a function of the normalized radial coordinate r, for $\eta = R/R_0 = 4\times10^{-3}$. The inset shows the evolution of the FisB flux at the neck radius $r = \eta$ over time.

NUCLEATION TIME OF FISB CLUSTERS AT THE NECK

To obtain a simple estimate of the nucleation time of a stable cluster of FisB proteins at the neck for both low-expression and native-expression strains, we assume that FisB proteins diffuse independently on the entire membrane and that nucleation of stable a cluster in the neck occurs when n proteins happen to be in the neck at the same time. To this end, we need to estimate the fraction of time there are n or more FisB proteins in the neck, as well as the correlation time, that is the time between uncorrelated samples. Since we assume FisB proteins are independent, the number of proteins in the neck will be Poisson distributed, thus we only need to know the average number of FisB in the neck to obtain the complete distribution. The average can be estimated as the neck area, $2\pi RL$, times the background concentration, ϕ_0. Furthermore, the correlation time is simply the time for a FisB to diffuse the length of the neck, L^2/D. Using the the values given in Table A, and taking the concentration $\phi_0 \simeq 20$ FisB μm^{-2} for the low-expression strain, yields $\langle\text{FisB}\rangle \simeq 0.03$ in the neck. Assuming that the ∼1-hour delay in membrane fission during sporulation observed in the low-expression strain is due to the time for nucleation, we can infer that the number of FisB proteins required for nucleation is $n = 3$, which yield a nucleation time of $t_N = \tau/\text{Pois}(3, \langle\text{FisB}\rangle) \simeq 60$ min. If the native-expression strain also needs $n \approx 3$ FisB proteins to nucleate, we can estimate its corresponding nucleation time using the value given in Table A, $\phi_0 \simeq 100$ FisB μm^{-2}, which yields $\langle\text{FisB}\rangle = 0.151$ and a nucleation time of $t_N \simeq 30$ s.

[1] P. B. Canham, J. Theor. Bio. **26**, 61 (1970).
[2] W. Helfrich, Z. Naturforsch. C. **28**, 693 (1973).
[3] O. Y. Zhong-Can and W. Helfrich, Phys. Rev. Lett. **59**, 2486 (1987).
[4] O.-Y. Zhong-Can and W. Helfrich, Phys. Rev. A **39**, 5280 (1989).
[5] U. Seifert and S. A. Langer, EPL **23**, 71 (1993).
[6] U. Seifert, Adv. Phys. **46**, 13 (1997).
[7] S. Leibler, J. Phys. **47**, 507 (1986).
[8] C. Zhu, S. L. Das, and T. Baumgart, Biophys. J. **102**, 1837 (2012).
[9] B. Sorre, A. Callan-Jones, J. Manzi, B. Goud, J. Prost, P. Bassereau, and A. Roux, Proc. Nat. Acad. Sci. U.S.A. **109**, 173 (2012).
[10] C. Tozzi, N. Walani, and M. Arroyo, New J. Phys. **21**, 093004 (2019).
[11] T. R. Powers, Rev. Mod. Phys. **82**, 1607 (2010).
[12] N. Ojkic, J. López-Garrido, K. Pogliano, and R. G. Endres, PLoS Comput. Biol. **10**, e1003912 (2014).
[13] B. Sabass and H. A. Stone, Phys. Rev. Lett. **116**, 258101 (2016).
[14] A. Karlsson, R. Karlsson, M. Karlsson, A. S. Cans, A. Strömberg, F. Ryttsén, and O. Orwar, Nature **409**, 150 (2001).
[15] P. Dommersnes, O. Orwar, F. Brochard-Wyart, and J. F. Joanny, EPL **70**, 271 (2005).
