# Supplementary material for: FisB relies on homo-oligomerization and lipid binding to catalyze membrane fission in bacteria
Source: PLoS Biol. 2021 Jun 29;19(6):e3001314. doi: 10.1371/journal.pbio.3001314 (PMC8274934; doi:10.1371/journal.pbio.3001314)
Supplement: Attachment — Submitted filename: ResponseToReviewers_210306.pdf [file pbio.3001314.s014.pdf]

We thank the reviewers for their constructive comments. We have revised our manuscript in light of their critiques. Our point-by-point replies to the reviewers' comments (in blue text) are provided below.

Reviewer #1:

The manuscript by Landajuela et al. examines FisB, a protein that catalyzes a critical membrane fission step during spore formation in the bacterium Bacillus subtilis. A previous report identified the protein, showed that it localized to the site of fission during sporulation, and that removal of the gene encoding the protein delayed the fission event. In this paper, the authors show in vivo that FisB clusters at the site of fission contain ~40 copies of FisB and experimentally confirmed the predicted topology of the protein in the membrane. They also isolated mutants of FisB that abrogated homodimerization and isolated point mutants in the extracellular domain of FisB that reduced electrostatic interactions with the membrane. Both FisB mutants displayed in vivo defects. Novelty aspects of the paper included results that showed that specific lipids or lipid microdomains are not required for localization of the protein to the site of fission. A main novel conclusion that the authors included in the abstract and introduction was that FisB likely recognizes the unique membrane topology at the site of fission. However, this model was only supported by a cross-species complementation experiment using a FisB ortholog that contained reduced sequence identity (23%, which is a significant level of identity; sequence similarity between the proteins may be even higher). The authors proposed that this result reduced the possibility that FisB recognizes another landmark at the site of fission, but the model was not directly tested. This was a straightforward continuing characterization of an important protein, but the strong conclusions of the main novelty aspect of the paper put forth in the abstract and intro were not directly tested (it is indeed a difficult model to test) and were not justified by the data. Specific comments are below.

Major comments:

1. The use of differential fluorescence intensity of TMA-DPH staining the forespore is apparently a new method for monitoring the completion of engulfment. Please report the "previous dye" (reference 23- presumably differential staining using a membrane-impermeable dye?) that was used, how that assay was different, and show the data demonstrating the similarity in kinetics of engulfment measured using both measurements.

The previous approach entailed use of the dye FM4-64, a method first reported by Sharp and Pogliano [1]. Since this dye is membrane-impermeable, cells that never entered sporulation and cells that have successfully undergone membrane fission at the end of engulfment both show the same labeling pattern: only the outer leaflet of the mother-cell membrane is labeled. To distinguish between these two cases another label is required, usually a forespore protein fused to CFP (PspoIIQ-CFP). Fig. S1B had already an example of labeling with the "previous dye" but this panel was not referenced, and the previous labeling method was not explained.

We have now modified the text in pp. 4-5 to explain the previous labeling strategy, referring to Fig. S1 (now referred to as S1 Appendix Figure 1), which we modified extensively by adding new results. Fig. S1B now includes an example of a cell that never entered sporulation, and which shows the same FM4-64 labeling as the one that has successfully completed engulfment

and has undergone membrane fission that was shown previously. New panel C compares the percentage of cells that have undergone membrane fission as a function of time after nutrient downshift detected using either method, as suggested. The two labeling approaches yielded indistinguishable results. New panels D-G explain how we detect membrane fission using the dye TMA-DPH and quantify the mean fluorescence intensities of the forespore contours.

2. I was unable to find how the authors defined the threshold fluorescence value of the forespore below which the authors conclude a cell has finished engulfment. Please report how "dim" a forespore must be to conclude that engulfment is finished.

We are sorry for this omission. We have added new panels D-G to Fig. S1 (now called S1 Appendix Figure 1) to show the difference in fluorescence intensities for cells that have or have not undergone fission (please also see our response above). The distributions of mean forespore contour intensities are clearly bimodal, with only a few percent of the cells with intermediate intensities that cannot be easily categorized. Because of this clear separation, in practice fission is usually scored visually. The intermediate cases are counted toward the total number of cells analyzed but are not considered in the percentages of cells that have or have not undergone fission. Thus, our estimates of percent membrane fission values are underestimated by a few percent. We note that with FM4-64 labeling we also see dim labeling of the forespore contours in a few percent of the cases; that is, results with FM4-64 also include a small fraction of cells that cannot be categorized.

In addition to Fig. S1, we also modified the text in pp. 4-5 and Material and Methods with this new information.

3. Fig. 1D, 1H. The authors state that "membrane fission is *always* accompanied by an" ISEP. Since Fig. 1H is only a correlation, and the authors in Fig. 1D directly monitor both fission and ISEP formation, please report the % coincidence of ISEP formation in cells that have dimmer forespores and report the number of cells counted.

Although it was clear that the vast majority of cells that had undergone membrane fission also had an ISEP, the actual fraction was not quantified. We now scored individual cells to quantify this fraction. As a result, we modified the passages in lines 165-166 and 174-175 as follows, respectively, to report the new information:

"Scoring individual cells, we found >90% (212/235) of cells that had undergone membrane fission also had an ISEP." (for the native expression levels).

"Scoring individual cells, we found >93% (258/277) of cells that had undergone membrane fission had an ISEP" (for the low-expression strain).

We removed *always* from the title of this subsection and throughout the text. We did similar quantifications for cells expressing the $FisB^{KK}$ mutant or the *C. perfringens* FisB which are now included in the modified text (lines 425-426 and line 455, respectively). In all cases, >90% of cells that had undergone membrane fission had an ISEP.

4. Fig. 4, S8. For clarity, the authors should consider distinguishing membrane binding of FisB (mediated by its hydrophobic N-terminus), from the *trans* membrane binding activity required for fission that I presume they are measuring in the liposome assay with

just the extracellular domain. As written, it was confusing to me why the authors conclude that a transmembrane protein's interaction with the membrane is "mainly electrostatic in nature".

In order to emphasize that the soluble extracytoplasmic fragment of the protein was used in these experiments, we have modified the passages (lines302, 320, and 762) referring to the experiments shown in Figs. 4 and S8 (which is now S1 Appendix Fig. 9). Additionally, we added a schematic of the soluble extracytoplasmic domain (ECD) of FisB used in the experiments shown in Fig. 4 to panel C. In S1 Appendix Fig. 9, panel A, we removed the domain structure for the full-length protein and left the one for the actual construct used in the experiments reported therein to avoid confusion.

Finally, we have added new experimental and modeling results that suggest "*trans*" interactions (bridging of membranes) are important for proper localization of FisB to the membrane neck where fission occurs in the cell (new sections "FisB does not have a preference for highly curved membrane regions, but can bridge membranes" and "Modeling suggests self-oligomerization and membrane bridging are sufficient to localize FisB to the membrane neck" (pp.12-15), new Figure 9, and new S2 Appendix: Theoretical Modeling).

We hope the constructs used in the liposome experiments and what is meant by "*trans*" interactions are clearer now.

5. Optional: Fig. 5-6. Does restoration of the KK motif elsewhere in the extracellular domain restore membrane association in vitro and/or fission in vivo?

We thank the reviewer for this is interesting suggestion. After some consideration, we decided it would be difficult to interpret the results of such experiments and that we should focus our efforts on experiments and modeling that might provide more substantial support to the idea that FisB recognizes the unique membrane topology at the site of fission.

6. FisB cross-species complementation. The results are straightforward, but the interpretation that protein-protein interactions may be ruled out by this test is far too strong and difficult to test. I recommend removing this conclusion here and stating only that a common localization landmark is likely recognized (membrane topology is certainly a possibility). I also recommend modifying the last sentence of the intro ("This idea is supported by complementation of B. subtilis ΔfisB cells by C. perfringens FisB, despite only ~23% identity between the two proteins.") to soften the conclusion.

We have followed the reviewer's suggestion and softened our interpretation of the cross-species complementation experiments throughout the manuscript.

7. The conclusion in the last paragraph of the introduction (also stated in the abstract) that the "requirements for FisB's sub-cellular localization" were determined was not supported by the data, since the influence of membrane topology was not directly tested in this report (it was only one possibility that the authors did not rule out, after testing many others). I recommend omitting these conclusions in the intro and in the abstract and only raising the membrane topology possibility in the Discussion section.

We now provide new experimental and modeling support for a mechanism by which FisB localizes to the membrane fission site. Specifically, new experiments show that FisB does not "sense" or generate membrane curvature but can bridge and aggregate artificial membranes (new Fig. 8 and new section "FisB does not have a preference for highly curved membrane regions but can bridge membranes"). Using new modeling, we show that this membrane bridging activity and self-oligomerization drive accumulation of FisB at the membrane neck where fission ultimately occurs (new Fig. 9, section "Modeling suggests self-oligomerization and membrane bridging are sufficient to localize FisB to the membrane neck", and new S2 Appendix: Theoretical Modeling). That is, FisB does utilize geometric cues for its sub-cellular localization but through a novel mechanism distinct from curvature-sensing. We have modified the rest of the manuscript accordingly, in particular the Introduction and Discussion.

Minor comment:

1. Results section, Fig. 1. Please briefly explain construction of the strain that produces less FisB in the main text so that that the reader need not look up reference 23.

Lower FisB expression was achieved by reducing the spacing between the ribosome binding site (RBS) and the ATG start codon, following ref. [2]. This information is now included on p.5 line 168-169.

Reviewer #2:

This is an elegant study that seeks to unravel the molecular determinants of FisB localization at the topologically unique tubular membrane intermediate formed during forespore fission in sporulating bacteria. This study is well-executed, technically thorough, and convincingly rules out a number of factors previously assumed to be determinants of FisB localization including CL interactions, PE (and associated negative membrane curvature), lipid microdomains etc. through well-controlled experiments. What is made clear through the use of varied mutants is that acidic lipid binding (presumably PG in vivo) as well as FisB self-assembly into higher-order structures are essential for FisB recruitment to this membrane neck and its role in fission. However, what retains FisB at this unique membrane structure is largely speculated upon rather than experimentally addressed.

We thank the reviewer for the comments. We have now added new experimental and modeling results to the manuscript in support of a mechanism that explains how FisB is recruited to the membrane fission site. Please see our reply to question #7 of Reviewer 1.

Here are my two major addressable concerns:

1. PG is postulated to be lipid that FisB interacts with at this membrane neck, which presumably helps retain FisB there. If this were true, then increasing PG content (versus CL or PE) in the membrane should increase the rate of formation of ISEP and of fission. Is this true? How is this lipid sequestered at the fission site (curvature sorting likely does not apply for PG)? Do FisB oligomers migrate with bound PG there? Lipid depletion experiments should be complemented by supplementation experiments.

We thank the reviewer for the suggested experiments. We had already increased the PG content at the expense of CL and PE but had not seen any detectable changes in FisB dynamics or membrane fission kinetics (Fig. 3). This is likely because negatively charged lipids (PG or CL)

are always abundant in the cell and do not limit FisB's localization. PG is already the major phospholipid in *B. subtilis* membranes (Fig. 3B, and ref. [3, 4]). In the *ΔclsABC, ΔpssA* mutant, PG is essentially the *only* detectable phospholipid (Fig. 3B, 4th lane). We conclude that PG cannot act as a reliable landmark, since it is everywhere!

How does FisB recognize the membrane neck connecting the engulfment membrane with the rest of the mother cell then? We present new experimental and modeling results that suggest membrane bridging and oligomerization of FisB drive its accumulation at the membrane fission site due to the neck geometry there (please also see our reply to comment #7 of Reviewer 1). We have added new sections (pp. 12-15), a new figure (Fig. 9), and new supplementary material describing the new experiments and model and modified the text elsewhere accordingly.

2. If the unique geometry of the prefission membrane intermediate is the overriding factor in FisB retention, then in principle, any self-assembling molecule that binds acidic lipids should be retained at the neck? Could the ECD of FisB be swapped with an unrelated domain that binds acidic lipids?

Unfortunately, any self-assembling molecule that binds acidic lipids is not necessarily retained at the membrane neck. An example is the GIII mutant which retains some self-oligomerization capacity (Fig. 5) yet is completely crippled in function (Fig. 6). Thus, it is likely that the strengths of the homo-oligomerization and membrane binding interactions need to be tuned to be within a certain range. New modeling provides some insights to the relevant parameters needed for the localization mechanism, including the strength of homo-oligomerization interactions (Fig. 9B).

We are not currently aware of any good candidate proteins that have all the required properties that could substitute for FisB, except for FisB homologs from other sporulating species (Fig. 7). In addition, we have often run into difficulties when trying to express FisB mutants (S1 Appendix Fig. 11), possibly because of their propensity to aggregate. Thus, although we agree it would be spectacular to find an artificial substitute based on the lipid-binding and self-oligomerization properties of FisB, this would be a challenging task well-beyond the scope of the current manuscript.

Curvature sensitivity and diffusion characteristics of FisB should be addressed using model membranes (e.g. using GUVs and tube pulling experiments). Does it prefer positive curvature over negative curvature that could explain its lipid preference? Or, in other words, how does curvature sort FisB localization or self-assembly? These will have to be experimentally addressed for a greater impact of the findings.

Otherwise, this is an excellent study at the cutting edge of its field.

We thank the reviewer for this excellent point. A related issue was raised by Reviewer #1. In response, we have carried out three independent series of experiments to test whether FisB has any preference for curved membranes (new Fig. 8 and section "FisB does not have a preference for highly curved membrane regions, but can bridge membranes").

Overall, these new experiments show that FisB does not use any intrinsic membrane curvature preference, but it can bridge membranes. Using this new information, we modeled how FisB might localize to the membrane neck where fission occurs. The model is described in new section "Modeling suggests self-oligomerization and membrane bridging are sufficient to

localize FisB to the membrane neck", new Fig. 9, and new S2 Appendix: Theoretical Modeling. In brief, modeling supports the idea that FisB can be recruited to the membrane neck via oligomerization without curvature sensing, and that an accumulation of FisB at the neck can be energetically stable. To the best of our knowledge, this is a novel sub-cellular localization mechanism that relies on the geometry generated during sporulation, yet is independent of curvature sensing.

Reviewer #3:

In this report, Landajuela et al. present a comprehensive set of experiments examining the mechanism by which the sporulation protein FisB catalyzes the process of membrane in fission in the bacterium Bacillus subtilis. Previous work by the same authors had already demonstrated that FisB played a key role in that process, but important aspects of the mechanisms remained unclear. Here, the authors tested multiple models and were able to exclude most of them, including the involvement of specific lipids such as cardiolipin, negative membrane curvature at the cell poles and the formation of lipid rafts. The careful and systematic approach that the authors followed in their investigation was necessary because deletion of fisB only has a modest effect on sporulation, implying that FisB is not fully essential for fission. Furthermore, it does not seem that additional sporulation proteins are required to interact with FisB. The authors successfully used a novel approach relying on DNA-origami calibration standards in fluorescence microscopy experiments with mGFP-FisB fusion proteins to measure the number of FisB molecules that aggregated in two types of clusters (i.e., mobile clusters of ~12 molecules and immobile cluster of ~40 proteins) that are formed at successive stages of the developmental process. They were thus able to propose that homo-oligomerization of FisB was a key factor in the mechanism of membrane fission. They also obtained and characterized homo-oligomerization mutants (where exposed hydrophobic residues are mutated). Next, they used a liposome co-flotation assay to investigate FisB binding to lipids and determined that the nature of the interaction was electrostatic and required a positively charged pocket in FisB and acidic lipids, i.e., the lipids did not need to be of a specific composition if they were negatively charged. Here again, they were able to generate mutants of FisB that were impaired in acidic lipids binding and showed that lipid-binding properties were independent from self-oligomerization. In summary, the work presented here is novel, of general interest and well designed; however, the following points should be addressed:

It is unfortunate that the authors forgot to add line numbers in their manuscript, so I will only be using page numbers in my comments.

We regret the omission of line numbering in the original submission. We have now added line numbers to the revised manuscript.

The term "membrane scission" is used in the title but never again in the rest of the paper where the term "membrane fission" is preferred. Are the two terms considered to be synonymous? If so, maybe "membrane fission" should be used in the title as well. To avoid repetition, the title could be rephrased as "FisB is dependent on homo-oligomerization and lipid-binding to catalyze membrane fission in bacteria"

We thank for the observation and the suggested title change. Indeed, we consider membrane "fission" and "scission" to be synonymous but did not realize scission was only used in the title. We changed the title to: "FisB relies on homo-oligomerization and lipid-binding to catalyze membrane fission in bacteria".

p.3, Ref.27 is highly controversial in the sporulation field. It would be better to replace it with a reference suggesting a more conservative estimate of spore longevity.

We have now replaced ref. 27 with a reference by Ulrich et al. (PLoS One, 2018) [5].

p.3, The dependency on SigE (and modest sporulation defect upon deletion of the gene) was reported earlier than ref. 23 (when the fisB gene was still known as yunB, PMID: 12662922)

We thank the reviewer for this correction. We have replaced ref. 23 with Eichenberger et al., J. Mol. Biol., 2003 [6] (lines 80-82).

p.4, Modification of the labeling strategy used in ref 40. Unless they are familiar with ref. 40, I am guessing that most readers will fail to grasp how the labeling strategy has been modified. Ref. 40 was using two membrane stains, one permeable (mitotracker green) and one impermeable (FM4-64) to the membrane barrier. I understand why MTG was deemed dispensable, but an explanation about why TMA-DPH was used instead of FM4-64 would be helpful.

We agree. The first comment of Reviewer #1 was closely related to this issue as well. In response, we have modified Fig. S1 and the main text (please also see our response to Reviewer #1).

p.4-5, Fig. 1D-E. It would be better if the authors were showing the results for mGFP (top row) in black and white rather green over black, as the contrast would be much better. This is especially important for the dimmer clusters that are hard to see. Also, in the cell highlighted as a representative example at hour 2, the clusters appear to be regularly spaced. Since this is a 2D image, is it possible that some clusters are connected and form continuous structures (e.g., rings) in 3D?

We have tried the reviewer's suggestion, which resulted in better distinction of mGFP spots from background. We also tried inverting the gray values, which results in dark spots on a light background. We found this representation is visually the best one. Hence, we modified Fig. 1D,E with this new representation.

Indeed, it appears that one of the cells shown in Fig. 1D has regularly spaced small mGFP clusters. However, this is usually not the case, as can be seen in other cells in the same image. Importantly, live imaging and tracking of clusters both in wide-field and total internal reflection fluorescence microscopy (TIRFM) indicate that the clusters move around independently (e.g. see S1 Appendix movie 1). Thus, it is highly unlikely that the clusters are connected and form continuous structures.

p.5, What is the cause for the lower expression of FisB in strain BAL003? I guess that the answer to this question can be found in ref. 23, but a brief reminder would be appreciated by the readers who are not fully familiar with the previous work.

Reviewer #1 had a similar question. Please see our response to Minor issue #1 of Reviewer 1.

p.5, tracking of DMC to estimate how rapidly they move. Unclear which dataset was used for this measurement. Is this information extracted from movies included in the Supplementary Information? If yes, please indicate so. Also, the acronym MSD is only defined in the figure legend but not in the text.

To clarify which data set was used for which measurement, we now state which S1 Appendix movie is related to which data set (S1 Appendix, Supplementary Movies). The movies provide examples of recordings and do not represent the entire data set. In most cases, to emphasize sub-cellular movements and to avoid very large files, we cropped out a single cell from a larger field of view. We now include more details about data sets that were analyzed in the text, figure legends, or both. For example, the legend of Fig. 2E now reads (the newly added information is highlighted):

E. Mean-squared displacement (MSD) as a function of delay time for DMCs (magenta) and ISEPs (blue). Cells expressing mGFP-FisB (strain BAM003) were imaged using time-lapse microscopy. Forty-five cells from 10 different movies at t=2.5 hr and 30 cells from 10 different movies at t=3 hr after nutrient downshift were analyzed. (See S1 Appendix Movie 1 for a representative single bacterium at t=2.5 hrs showing several mobile DMCs and Movie 2 for a representative single bacterium at t=3 hrs showing an immobile ISEP.) Fits to the initial 25 s (~10 % of delays) yielded $D_{DMC} = 2.80 \pm 0.05 \times 10^3\ \mathrm{nm}^2/\mathrm{s}$ (± 95% confidence interval, R^2 = 0.999, 24 tracks) and $D_{ISEP} = 2.80 \pm 0.51 \times 10\ \mathrm{nm}^\wedge 2/\mathrm{s}$ (± 95% confidence interval, R^2 = 0.850, 25 tracks).

We now define the acronym MSD in the text where it first appears (p. 6, line 204).

p.13, Although it cannot be entirely excluded that genetic differences between the PY79 and 168 strains could account for the discrepancy with the results of Kawai et al, this explanation seems unlikely. The genetic differences between 168 and PY79 are well documented and unlikely to affect sporulation properties. Besides, it would be relatively easy to conduct a series of control experiments in the 168 genetic background. Nevertheless, I do not think that it is necessary to carry out these experiments as they are not critical for the proposed model.

We thank the reviewer for the insight. An alternative explanation for the discrepancy is that our detection sensitivity may be somewhat lower than that of Kawai et al., as slightly different protocols were used for the detection of phospholipids in the two cases. We therefore changed the passage (lines 684-686) to: "We suggest the differences may be due to the different strains used[84], PY79[85] here vs. BS168[86] in Kawai et al. or differences in detection sensitivities."

1. Sharp, M.D. and K. Pogliano, *An in vivo membrane fusion assay implicates SpoIIIE in the final stages of engulfment during Bacillus subtilis sporulation.* Proc Natl Acad Sci U S A, 1999. **96**(25): p. 14553-8.
2. Doan, T., et al., *Novel secretion apparatus maintains spore integrity and developmental gene expression in Bacillus subtilis.* PLoS Genet, 2009. **5**(7): p. e1000566.
3. den Kamp, J.A., I. Redai, and L.L. van Deenen, *Phospholipid composition of Bacillus subtilis.* J Bacteriol, 1969. **99**(1): p. 298-303.
4. Kawai, F., et al., *Cardiolipin domains in Bacillus subtilis marburg membranes.* J Bacteriol, 2004. **186**(5): p. 1475-83.
5. Ulrich, N., et al., *Experimental studies addressing the longevity of Bacillus subtilis spores - The first data from a 500-year experiment.* PLoS One, 2018. **13**(12): p. e0208425.
6. Eichenberger, P., et al., *The sigmaE regulon and the identification of additional sporulation genes in Bacillus subtilis.* J Mol Biol, 2003. **327**(5): p. 945-72.
