# Supplementary material for: FisB relies on homo-oligomerization and lipid binding to catalyze membrane fission in bacteria
Source: PLoS Biol. 2021 Jun 29;19(6):e3001314. doi: 10.1371/journal.pbio.3001314 (PMC8274934; doi:10.1371/journal.pbio.3001314)
Supplement: Attachment — Submitted filename: Response_Referee4.pdf [file pbio.3001314.s016.pdf]

RESPONSE TO REVIEWER 4

To address the concerns raised by the previous round of reviews, the authors adding a physical model to explain how the trans-aggregation of FisB can be energetically favorable. I have the following comments for the authors to consider. Overall, I think the modeling section should be written with more clarity to better serve its purpose in this paper. There are parts that are quite confusing preventing the reader from fully appreciating the value proposition that the model makes.

First and foremost we would like to thank the referee for carefully reading our paper and for the constructive suggestions. We believe that the changes introduced thanks to the reviewer's comments have substantially improved the modeling section, especially in terms of clarity. In the revised manuscript changes are highlighted in blue. In the following, we include our responses to the comments and suggestions made by the referee.

1. **The free energy of the membrane is written as a bending energy plus the energy of membrane-protein interactions. What is interesting is that there are two separate membranes that are trying to fuse together to complete the fission process and this fusion of these two membranes is mediated by these trans interactions. I ask because from all the schematics (Fig 1A i to Fig 9 and the supplementary material figures), this is what is depicted. Since the membrane is assumed to be locally flat and uncoupled from the curvature, why is the bending energy necessary? I feel like I'm missing some explanation that should be clarified in what energy is being minimized (literally — what is the system and what are the surroundings?). I wonder if the issue can be resolved by writing the energies of membrane domain 1, membrane domain 2 and the membrane-protein adhesion energies for clarity but perhaps the authors have better ideas.**

   We regret that the reviewer may have misunderstood the intended model geometry, likely due to our very simplified schematics. We apologize for this lack of clarity. In particular, the simplified geometry we are considering in our model is an axisymmetric cylindrical neck/pore connecting two planar membranes, which aims to reproduce the local region where the engulfment membrane meets the rest of the mother cell membrane. For this

reason, there is a bending energy associated with the cylindrical neck but not with the approximately planar membranes connected by the neck. We have clarified this geometry by changing the schematics in the main text and in the supplementary material. We include the updated figure below.

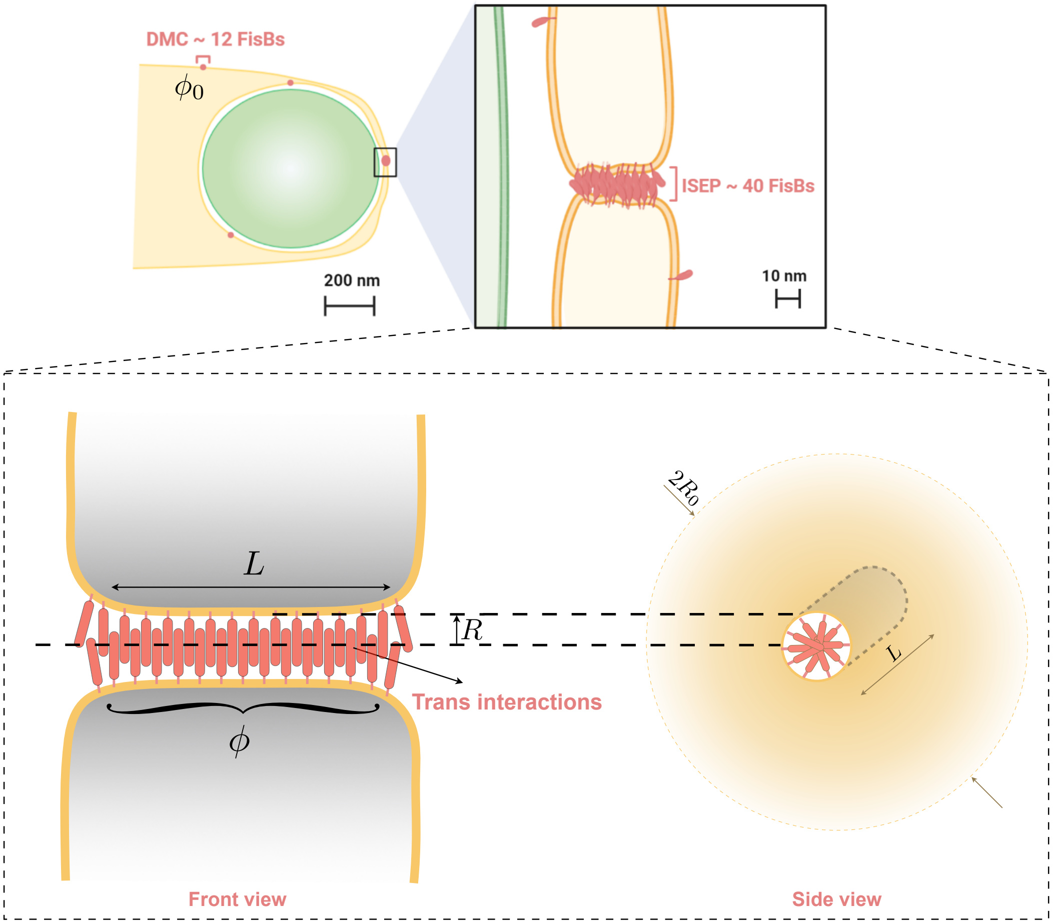

2. **So if it is two separate membranes trying to fuse, then aren't fluctuations and the local hydrodynamics important? Can the authors comment on the role these factors may play?**

We thank the referee for raising this important issue. Thermal fluctuations as well as hydrodynamics will certainly be important during membrane fission. However, in the present work, our aim was only to model the formation of a stable FisB cluster at the neck prior to fission. To this end, we took into account only the essential protein-protein interactions and a simplified version of the geometry within a mean field model. The coupling between membrane dynamics and hydrodynamics involved during fission surely deserves further study.

We have included a sentence in both the main and SI texts to explicitly state the relevance of these issues, especially during fission, but which are beyond the scope of the present work. Indeed, we are currently working along the suggested lines.

3. **Essentially, the authors are using a LJ potential and a repulsion energy $U(\phi)$ to capture the adhesion interactions of the trans-FisB molecules and minimize the cis interactions. This is fine but again, why not cite the relevant references that have actually considered this problem at various levels of complexities? I might add that this is true of the entire modeling section — it is rather sparse on references in the field, even missing the seminal ones that have agonized about fusion etc.**

   We thank the referee for this comment and we apologize for not having included relevant references using similar protein-protein interaction potentials. We have now included appropriate references that consider similar energy functionals [1–4].

4. **Boundary conditions — the authors should mention that role that boundary conditions might play in altering the energy minimization. No mention is made of the edge effects, which are going to be critical in determining the neck.**

   We thank the reviewer for raising this relevant point. As the reviewer states, boundary effects might play an important role in determining the equilibrium radius. We actually explored the complete minimization problem where the shape of the neck $h(z)$ and the FisB density $\phi(z)$ are obtained as functions of the axial coordinate z by solving the complete Euler-Lagrange system of equations:

$$0 = -\gamma_{\text{eff}} H + \partial_h V_{\text{LJ}}(h) h \sqrt{1+(\partial_z h)^2} + \kappa \left(\frac{H^3}{2} - 2HK + \nabla_s^2 H \right), \tag{1}$$

$$k_{\text{B}} T [1 + \ln(\phi/\phi_0)] + 2a\phi V_{\text{LJ}}(h) + \partial_\phi U(\phi) = 0, \tag{2}$$

   where $\gamma_{\text{eff}} = \gamma + k_{\text{B}} T \phi \ln(\phi/\phi_0) + a\phi^2 V_{\text{LJ}}(h) + U(\phi)$ and ∇_s is the Laplace-Beltrami operator. Solving the above equations actually yields very similar results, thus the main conclusions of our simplified model remain the same, and it has the advantage of a more accessible formulation.

   Additionally, the equilibrium radius in the presence of FisB proteins is significantly smaller than the observed length of the neck. Therefore, in this scenario, we expect that the boundaries do not play a significant role in the minimization problem since $L \gg R$, and thus

$\Delta L \sim R \ll L$, ΔL being the characteristic length of the boundaries, where all the terms in Eq. (1) would become important. Indeed, the dimensionless parameter that characterizes the role of the boundaries in our model is β, which is relatively small according to the estimates of Table A, namely $\beta \sim 10^{-1}$. Therefore, we expect only a small contribution from the boundaries to the minimization problem. In the SI, we have included a discussion along these lines to justify this simplification.

5. **notation — what are σ and a? I couldn't find any explanation for all the notation used. Also, much of the supplement and the modeling section are near verbatim in many paragraphs. Please edit to remove repetition.**

   We thank the reviewer for this comment. We have now explicitly defined σ and a in words, both in the main text and the SI. In particular, σ is the characteristic length of the LJ potential associated with protein-protein trans interactions, and a is the trans-interaction energy per FisB density.

   Concerning repetition, our intention was to write a self-contained supplementary material, but as the reviewer states, we have repeated a few paragraphs and sentences. We have rephrased some of these for greater clarity and to avoid repetition.

6. **One other curiosity is that the authors predict a radius of 2.7 nm, which is roughly half the thickness of the bilayer and well below the applicability of the Helfrich model. Can the authors comment on the limitations of their model?**

   We thank the referee for raising this important issue. We are aware that we have pushed the Helfrich continuum model to the limit by obtaining an equilibrium radius of ~ 3 nm. This specific value is mainly due to our choice, based on the known structure of FisB, of the value of σ which controls the minimum of the trans-interaction potential, and thus the equilibrium radius R. Indeed, we could have obtained a larger equilibrium radius by using a larger value of σ, but this would be equally approximate, since we do not yet have precise experimental measurements of the neck radius. Hence, for simplicity, we chose σ to be the same as in the FisB-FisB cis interactions, which yields $R \sim 3$ nm. We have included a comment along these lines in the main text and the SI. Additionally, there are some theoretical works, namely Refs. [4, 5], which also obtain equilibrium radii of similar magnitude using the

continuum Helfrich model.

7. **section on diffusion — shouldn't the Laplacian operator be a Laplace-Beltrami operator on the surface? Also, in the absence of large curvatures and curvature-mediated interactions, why would diffusion be considered rate limiting? This is also another section that is missing many references.**

   In the section on diffusion we analyze the time needed for FisB monomers in the surrounding planar membranes to diffuse and be captured at the neck. Since the curvature of these surrounding membranes is assumed to be negligible, the Laplace-Beltrami operator is not required. We apologize for any confusion, and we believe that with the new schematic figures (reproduced below) this issue is clarified. The calculation of the rate of diffusion is simply intended to demonstrate that diffusion is fast enough not to be rate limiting. To clarify this point, we now state both in the main text and SI that the rate limiting process is nucleation and not diffusion.

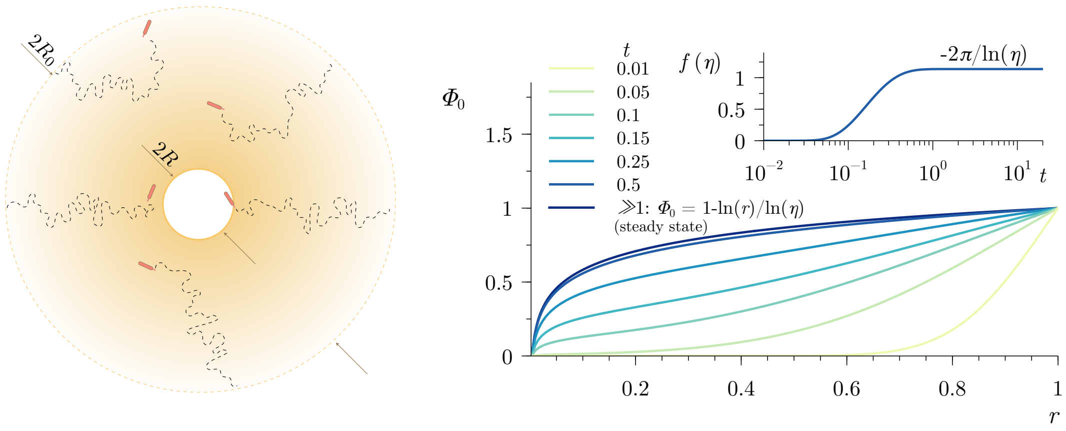

---

[1] S. Leibler, J. Phys. **47**, 507 (1986).

[2] C. Zhu, S. L. Das, and T. Baumgart, Biophys. J. **102**, 1837 (2012).

[3] B. Sorre, A. Callan-Jones, J. Manzi, B. Goud, J. Prost, P. Bassereau, and A. Roux, Proc. Nat. Acad. Sci. U.S.A. **109**, 173 (2012).

[4] C. Tozzi, N. Walani, and M. Arroyo, New J. Phys. **21**, 093004 (2019).

[5] B. Sabass and H. A. Stone, Phys. Rev. Lett. **116**, 258101 (2016).
