# Supplementary material for: Correction: FisB relies on homo-oligomerization and lipid binding to catalyze membrane fission in bacteria
Source: PLoS Biol. 2021 Dec 14;19(12):e3001499. doi: 10.1371/journal.pbio.3001499 (PMC8670662; doi:10.1371/journal.pbio.3001499)
Supplement: S1 Appendix — (DOCX) [file pbio.3001499.s001.docx]

**S1 Appendix: Supplementary Experimental Information**

**FisB relies on homo-oligomerization and lipid-binding to catalyze membrane fission in bacteria**

**Ane Landajuela^1,2†^*, Martha Braun^2,3†^, Christopher D. A. Rodrigues^4^, Alejandro Martínez-Calvo^5^, Thierry Doan^6^, Florian Horenkamp^7#^, Anna Andronicos^1‡^, Vladimir Shteyn^1,2,^ Nathan D. Williams^2,7^, Chenxiang Lin^2,7^, Ned S. Wingreen^8,9^, David Z. Rudner^10^, Erdem Karatekin^1,2,3,11*^**

1 Cellular and Molecular Physiology, Yale University, New Haven, CT, USA

2 Nanobiology Institute, Yale University, West Haven, CT, USA

3 Molecular Biophysics and Biochemistry, Yale University, New Haven, CT, USA

4 ithree Institute, University of Technology Sydney (UTS), Australia

5 Grupo de Mecánica de Fluidos, Universidad Carlos III de Madrid, Spain

6 Laboratoire d’Ingénierie des Systèmes Macromoléculaires, Aix-Marseille Université - CNRS UMR7255, Marseilles, France

7 Cell Biology, Yale University

8 Department of Molecular Biology, Princeton University, Princeton, NJ 08544, USA.

9 Lewis-Sigler Institute for Integrative Genomics, Princeton University, Princeton, NJ 08544, USA.

10 Department of Microbiology, Harvard Medical School, Boston MA

11 Université de Paris, SPPIN - Saints-Pères Paris Institute for the Neurosciences, Centre National de la Recherche Scientifique (CNRS), F-75006 Paris, France.

† These authors contributed equally

# Present address: Pfizer, New London, CT

**^‡^** Present address: Department of Biological Chemistry, School of Medicine, U. California Irvine

* To whom correspondence should be addressed

ane.landajuela@yale.edu

erdem.karatekin@yale.edu (lead contact)

**Quantification of FisB copy numbers using *B. subtilis* fluorescence standards and Western blotting.**

We used two independent approaches to quantify FisB copy numbers in addition to the DNA-origami based quantification presented in the main text. First, we used *B. subtilis* calibration strains^1^ to relate the total cell fluorescence (sum of pixel values) to copy numbers of super-folder GFP (sfGFP). Cells expressing known copy numbers of sfGFP were imaged in wide-field fluorescence and the total intensity, corrected for background, was plotted against sfGFP copy number (S1 Appendix Fig. 2A,B). We then imaged cells expressing an equivalent number of sfGFP (strain SG13) or mEGFP (strain BAL038) molecules and found the total fluorescence was 2.4±0.2-fold higher for sfGFP (S1 Appendix Fig. 2C). Using this correction factor, the calibration for sfGFP was converted to a calibration for mEGFP, the label used for imaging FisB (S1 Appendix Fig. 2D). We then computed the distribution of total cell fluorescence in *B. subtilis* *ΔfisB* cells expressing mEGFP-FisB (BAL001) at native levels at t=3 h into sporulation (S1 Appendix Fig. 2E). We also estimated the total fluorescence of DMC and the ISEP as a fraction of total cellular fluorescence intensity (S1 Appendix Fig. 2F). Together with the calibration of S1 Appendix Fig. 2D, the means of these distributions allowed us to estimate ~1300 total FisB copies per cell, of which ~50 are located at the ISEP. The DMCs contain ~16 copies of FisB on average by this estimate.

Second, we used quantitative Western blotting (WB) to relate the total number of FisB molecules per cell to fluorescence intensity. Purified recombinant mYFP was used to calibrate the average number of mGFP-FisB molecules per cell using WB, using anti-GFP antibodies (S1 Appendix Fig. 3). This average number per cell (~700) was then used to calculate mGFP-FisB copies at the ISEP (~30 copies) and DMC (~10 copies) from the fluorescence measurements in S1 Appendix Fig. 2F, the fraction of total cellular fluorescence signal found at ISEP or DMC.

**Localization of FisB is not coupled to cell wall remodeling, the protonmotive force, or the membrane potential**

The sub-cellular localization and motion of many cellular components depend on the cell-wall remodeling machinery^2-4^, the protonmotive force and the membrane potential^5^. We tested if any of these influenced dynamics of FisB.

Cell wall synthesis and degradation drive engulfment during sporulation^2,6^. It was suggested that cell wall remodeling might also drive fission of the engulfing membrane at the end of engulfment. We wondered whether FisB dynamics could be coupled to cell wall remodeling. Since inhibition of cell wall synthesis leads to engulfment defects, we expressed mGFP-FisB from an inducible promoter during vegetative growth and investigated effects of inhibition of cell wall synthesis by fosfomycin on the motion of FisB clusters. As a control, we chose to image GFP-Mbl in parallel experiments. Mbl is an actin homologue that controls cell wall synthesis and cell shape^7^. Mbl forms filaments that are associated with the cell membrane and rotate around the cell circumference together with enzymes required for cell wall synthesis^8^. Mbl filaments stop moving and eventually disassemble upon treatment with fosfomycin^9^ which inhibits the formation of N-acetylmuramic acid, a building block of the bacterial cell wall^10^.

We imaged cells expressing either mGFP-FisB (BMB014) or GFP-Mbl (BDR2061) using total internal reflection fluorescence microscopy (TIRFM) in the absence and presence of fosfomycin (S1 Appendix Fig. 6). In this imaging mode, only fluorophores within ~100 nm of the glass-aqueous buffer interface are detected^11^. That is, only the spots near the substrate-proximal side a cell would be visible. Small mGFP-FisB spots, similar to the ones present during early stages of engulfment, moved around in the cell membrane seemingly randomly (S1 Appendix Movies 3 and 4). By contrast, GFP-Mbl spots moved along the short axis of the cell (S1 Appendix Movies 5 and 6), as previously reported^8,12^. To quantify these motions, we used four different approaches. First, we computed kymographs of mGFP-FisB or GFP-Mbl along the short and long axes of a cell, as shown in S1 Appendix Figure 6A, C. Before treatment, GFP-Mbl moved around the cell circumference, reflected by stripes across the cell in the maximum intensity projections (MIP) and spots that appear and disappear in the kymographs along the long axis (marked with a red frame). Spots also appear and disappear along the short axis as GFP-Mbl spots move in and out of the evanescent field as they move along the cell circumference (kymographs marked by blue frames). Addition of fosfomycin stopped the motion of GFP-Mbl (S1 Appendix Movies 7 and 8), resulting in small spots in the MIP and continuous lines in the kymographs. By contrast, mGFP-FisB MIPs and kymographs were not appreciably modified upon fosfomycin treatment (S1 Appendix Movies 9 and 10). Second, we tracked individual GFP-Mbl and mGFP-FisB spots and calculated the mean-squared displacement (MSD) (S1 Appendix Figure 6B,D). Addition of fosfomycin reduced the motility of GFP-Mbl filaments (S1 Appendix Figure 6B), whereas the motion of mGFP-FisB was unaffected (S1 Appendix Figure 6D). Third, the average total distance that GFP-Mbl filaments traveled within 3 s was reduced in the presence of fosfomycin, whereas no such effect was found for the distance traveled by mGFP-FisB spots (S1 Appendix Figure 6E). Finally, we computed the asymmetry^13^ of the individual GFP-Mbl and mGFP-FisB trajectories, and computed averages (S1 Appendix Figure 6F). Asymmetry is a measure of the tendency for a persistently preferred direction of motion: asymmetry is zero for a perfectly symmetric trajectory, whereas it diverges for a straight-line trajectory. For simulated 2-dimensional Brownian trajectories, asymmetry rapidly converges to ~0.26 for a large number of steps and/or particles. The asymmetry of GFP-Mbl trajectories before fosfomycin treatment was high, equal to $0.58 \pm0.06$ (mean ± SEM), consistent with previous reports of Mbl moving in linear tracks^8^. Upon treatment, GFP-Mbl spots stopped moving, and the asymmetry decreased to $0.30\pm0.07$. By contrast, the asymmetry of mGFP-FisB trajectories before ($0.22\pm0.03$) and after treatment ($0.23\pm0.06$) were similar (S1 Appendix Figure 6F). Thus, the motion of FisB clusters is independent of cell-wall synthesis.

The protonmotive force (PMF) is important for the localization of proteins that are involved in maintaining cell shape, such as MreB and Mbl, or cell division (e.g. FtsZ/FtsA)^5^. We tested whether the localization of FisB depends on the PMF by imaging mGFP-FisB (BAM003) during sporulation in the absence and presence of carbonyl cyanide m-chlorophenyl hydrazone (CCCP), a proton-ionophore that dissipates the membrane PMF. We found that the localization of GFP-FisB at t=3 h into sporulation is not affected by the PMF unlike the localization of Mbl (S1 Appendix Figure 6G).

Proteins whose localization depends on the PMF also require an intact membrane potential. To see if the membrane potential affected FisB dynamics, we imaged mGFP-FisB (BAM003) in the presence and absence of valinomycin, an antibiotic that functions as a potassium carrier that depletes the transmembrane electric potential component of the PMF. We found that GFP-Mbl mislocalizes in the presence of valinomycin, whereas the localization of FisB is not affected (S1 Appendix Figure 6G).

Together, these results show that the dynamic movement of FisB is independent of cell wall remodeling, the PMF, and the membrane potential.

**Topology of FisB**

The FisB protein family is defined through a consensus region (residues 130-223, Figure 4A) identified in Pfam^14^. Most algorithms predict FisB to possess a single transmembrane domain (TMD) with a small N-terminal cytoplasmic domain and a larger (23-kDa) extracellular domain (ECD), as depicted Figure 4A. However, some algorithms assign a hydrophobic region around residues 130-150 as a second TMD, or even predict an inverted topology (S1 Appendix Figure 7B). If there were a second TMD (S1 Appendix Figure 8C), or if the topology were inverted, the C-terminus would face the cytosol rather than the extracytoplasm. To determine FisB’s topology, we tested accessibility of cysteine residues introduced at various positions to a membrane impermeable, biotinylated, sulfhydryl-reactive reagent, 3-(N-maleimidylpropionyl) biocytin (MPB)^15^. We generated three Myc-tagged FisB mono-cysteine variants (Myc-tagged FisB G6C, FisB L137C and FisB A245C) and tested separately whether these cysteines were intra-or extracellular. When complementing ΔfisB cells, these mutations reduced the sporulation efficiency slightly (S1 Appendix Table 2), we assume without an effect on topology. We first tested cysteine accessibility to MPB in lysed protoplasts of sporulating cells. Membranes were solubilized with detergent and FisB was pulled down using a polyclonal anti-myc antibody. Biotinylation was probed by Western Blot using an HRP-conjugated-avidin antibody. This analysis showed that only residues 6 and 245 were accessible (S1 Appendix Figure 8D left panel, top row), suggesting residue 137 may be restricted by secondary/tertiary structures and/or the membrane. In intact protoplasts only residue 245 was labeled by MPB, indicating the C-terminus faces the extracellular space (S1 Appendix Figure 8D middle panel). In contrast, only residue 6 was biotinylated when extracellular cysteines were blocked by 4-acetamido-4′-maleimidylstilbene-2,2′-disulfonic acid (AMS) prior to cell lysis and incubation with MPB. That is, residue 6 faces the cytoplasm (S1 Appendix Figure 8D right panel). To confirm the presence of FisB, we stripped every Western Blot and probed it with anti-FisB antibody (S1 Appendix Figure 8D, bottom row). Together, these results are consistent with a topology in which the larger C-terminus of FisB is extracellular, the N-terminus faces the cytoplasm, and residue 137 is inaccessible to biotinylation, possibly residing inside a globular domain, shielded at the oligomerization interface, or by the membrane. Our attempts to determine the structure of FisB ECD were unsuccessful, but a computational model of FisB for residues 44 to 225, covering most of the ECD is available^16^ and is shown in Figure 4B. The model predicts a curved ECD structure, with ~3 nm and ~5 nm for the inner and outer radii of curvatures. The overall topology of FisB, with the predicted ECD structure is schematically shown Figure 4B.

**Table 1. Summary of FisB copy numbers per cell, ISEP and DMC in the native and low expression strains obtained using different calibrations**.

| **Calibration method** | ***B. subtilis* standards** | | **DNA origami** | | **Quantitative WB** | |
| --- | --- | --- | --- | --- | --- | --- |
| **Expression Level** | **low** | **native** | **low** | **native** | **low** | **native** |
| **Total copies of**  **m(E)GFP-FisB per cell** | **160**  **±66^a^** | **1300 ±500^c^** | **122**  **±51^f^** | **963**  **±376^h^** | **83**  **±6^n^** | **661**  **±49^k^** |
| **Copies at ISEP (t=3h)** | **8±2^b^** | **50±18^d^** | **6±2^g^** | **36±15^i^** | **5±3^o^** | **26±13^l^** |
| **Copies at DMC**  **(t=2h)** | **n.d** | **16±10^e^** | **n.d** | **12±7^j^** | **n.d** | **9±6^m^** |

**a.** Total FisB copies per cell (mean±SD) were calculated from the distribution shown in Fig. S4B (red) and the calibration using the B. subtilis calibration strains ($y=22.48x, R^{2}=0.93$, Fig. S2D). The error estimate is from the SD of the distribution in Fig. S4B. **b.** Calculated using mean±SD from the distribution shown in Fig. S4D (red) and the calibration with B. subtilis calibration strains, Fig. S2D. **c.** Calculated using mean±SD from the distribution shown in Figure S4B (green) and the calibration in Fig. S2D. **d**. Calculated using mean±SD of the distribution shown in Fig. S4D (green) and the calibration in Figure S2D. **e.** Calculated using mean±SD of the distribution shown in Fig. 2C (blue) and the calibration in Figure S2D. **f.** Calculated using mean±SD from the distribution shown in Fig. S4B (red) and the calibration obtained with DNA origami standards, Fig. 2D ($y=29.56x;R^{2}=0.97)$. **g**. Calculated using mean±SD of the distribution shown in Fig. S4D (red) and the calibration in Fig. 2D. **h**. Calculated using mean±SD of the distribution shown in Figure S4B (green) and the calibration in Fig. 2D. **i.** Calculated using mean±SD of the distribution shown in Fig. S4D (green) and the calibration in Fig. 2D. **j.** Calculated using mean±SD of the distribution shown in Fig. 2C (blue) and the calibration in Fig. 2D. **k**. Calculated from Western blot calibration using purified mYFP ($y=2783,R^{2}=0.98$, Figure S3A) together with the Western blots of mXFP-FisB using a known number of cells (Figure S3B). mXFP-FisB from $1.44\times{10}^{7}$B. subtilis cells loaded into the gel in Fig S3B produced a WB band intensity corresponding to $0.6\pm0.05$ ng ($6.61\pm0.49\times{10}^{9}$ molecules) in Fig. S3A. This was corrected for cleaved mXFP (~44%, Fig. S5B). **l**. Calculated using percentage of total cellular FisB located at ISEP estimated in Fig S2F and the total copies of mXFP-FisB per cell in k. **m**. Calculated as in l. **n.** Estimated by dividing FisB copies per cell (k) by 8. **o**. Calculated using mean±SD of the distribution of percentage cell o fluorescence located at ISEP in Figure S4E.

**Table 2. Bacillus subtilis strains used in this study.**

| **Strain** | **Genotype** | **% spo^a^** | **Source** |
| --- | --- | --- | --- |
| PY79 | *Prototrophic wild-type strain* | 100±8.36 | ^23^ |
| BDR1083 | *∆fisB::tet* | 13.17 ± 1.97 | ^24^ |
| BKM15 | *amyE::PspoIIQ-cfp (spec)* | n.d | ^25^ |
| BAM003 | *∆fisB::tet ycgO::PfisB-mGFP -fisB (cat)* | 90.17±12.65 | ^25^ |
| BVS001 | *∆fisB::tet ycgO::PfisB-mYFP -fisB (cat)* | n.d | This work |
| BAL001 | *∆fisB::tet ycgO::PfisB-mEGFP -fisB (cat)* | n.d | This work |
| BAL002 | *∆fisB::tet, ycgO::PspoIID-RBSfisB(5n)-mYFP A206K-fisB (erm)* | 83.54 ± 4.93 | This work |
| BAL003 | *∆fisB::tet, ycgO::PspoIID-RBSfisB(5n)-mGFP A206K-fisB (erm)* | n.d | This work |
| BAL004 | *∆fisB::tet, ycgO::PspoIID-RBSfisB(5n)-mEGFP A206K-fisB (erm)* | n.d | This work |
| BAL005 | *∆fisB::tet ycgO::PfisB-mEGFP-fisB Clostridum perfringens sp. (cat)* | 85 ± 5 | This work |
| BAL006 | *∆fisB::tet, ycgO::PspoIID-RBSfisB(5n)-mYFP A206K-fisB* K168D K170D *(erm)* | 16.91±7.03 | This work |
| BAL007 | *∆fisB::tet, ycgO::PspoIID-RBSfisB(5n)-mYFP A206K-fisB* G175A I176S I194T I195S *(erm)* | 3.03±2.51 | This work |
| BAL008 | *∆fisB::tet, ycgO::PspoIID-RBSfisB(5n)-mYFP A206K-fisB ∆80-96 (erm)* | 13.04±2.71 | This work |
| BAL009 | *∆fisB::tet, ycgO::PspoIID-RBSfisB(5n)-mYFP A206K-fisB ∆122-132 (erm)* | 99.42±15.03 | This work |
| BAL010 | *∆fisB::tet, ycgO::PspoIID-RBSfisB(5n)-mYFP A206K-fisB ∆137-154 (erm)* | 14.09±6.33 | This work |
| BAL011 | *∆fisB::tet, ycgO::PspoIID-RBSfisB(5n)-mYFP A206K-fisB ∆167-182 (erm)* | 16.70±6.99 | This work |
| BAL012 | *∆fisB::tet, ycgO::PspoIID-RBSfisB(5n)-mYFP A206K-fisB ∆210-220 (erm)* | 15.30±3.32 | This work |
| BAL013 | *∆fisB::tet, ycgO::PspoIID-RBSfisB(5n)-mYFP A206K-fisB ∆*132-222*(erm)* | 14.78±3.40 | This work |
| BAL014 | *∆fisB::tet, ycgO::PspoIID-RBSfisB(5n)-mYFP A206K-fisB* L90T ( erm) | 104.31±0.00 | This work |
| BAL015 | *∆fisB::tet, ycgO::PspoIID-RBSfisB(5n)-mYFP A206K-fisB L137S G138A (erm)* | 16.74±2.39 | This work |
| BAL016 | *∆fisB::tet, ycgO::PspoIID-RBSfisB(5n)-mYFP A206K-fisB L145TL146S (erm)* | 16.52±2.60 | This work |
| BAL017 | *∆fisB::tet, ycgO::PspoIID-RBSfisB(5n)-mYFP A206K-fisB G150A (erm)* | 23.91±3.04 | This work |
| BAL018 | *∆fisB::tet, ycgO::PspoIID-RBSfisB(5n)-mYFP A206K-fisB* V215S G217A *(erm)* | 10.43±1.73 | This work |
| BAL019 | *∆fisB::tet, ycgO::PspoIID-RBSfisB(5n)-mYFP A206K-fisB* V219T *(erm)* | 23.48±0.00 | This work |
| BAL020 | *∆fisB::tet, ycgO::PspoIID-RBSfisB(5n)-mYFP A206K-fisB* G175A I176S *(erm)* | 9.91±5.14 | This work |
| BAL021 | *∆fisB::tet, ycgO::PspoIID-RBSfisB(5n)-mYFP A206K-fisB* I194T I195S (erm) | 45.65±12.97 | This work |
| BAL022 | *∆fisB::tet, ycgO::PspoIID-RBSfisB(5n)-mYFP A206K-fisB*  R56E *(erm)* | 55.00±15.00 | This work |
| BAL023 | *∆fisB::tet, ycgO::PspoIID-RBSfisB(5n)-mYFP A206K-fisB* E67R D68K *(erm)* | 37.30±12.70 | This work |
| BAL024 | *∆fisB::tet, ycgO::PspoIID-RBSfisB(5n)-mYFP A206K-fisB* K106D K109D *(erm)* | 22.50±7.50 | This work |
| BAL025 | *∆fisB::tet, ycgO::PspoIID-RBSfisB(5n)-mYFP A206K-fisB* K116D *(erm)* | 65.70±14.30 | This work |
| BAL026 | *∆fisB::tet, ycgO::PspoIID-RBSfisB(5n)-mYFP A206K-fisB* E119R *(erm)* | 55.00±15.00 | This work |
| BAL027 | *∆fisB::tet, ycgO::PspoIID-RBSfisB(5n)-mYFP A206K-fisB* R156E *(erm)* | 65.50±22.50 | This work |
| BAL028 | *∆fisB::tet, ycgO::PspoIID-RBSfisB(5n)-mYFP A206K-fisB K170D K172DE* (erm) | 16.40±1.45 | This work |
| BAL029 | *∆fisB::tet, ycgO::PspoIID-RBSfisB(5n)-mYFP A206K-fisB* K192D *(erm)* | 55.16±14.80 | This work |
| BAM234 | *∆ywnE::cat ∆ywjE::kan ∆ywiE::erm* | 25.88 ± 8.53 | ^25^ |
| BAM236 | *∆fisB::tet ∆ywnE::cat ∆ywjE::kan ∆ywiE::erm* | 1.07 ± 0.52 | ^25^ |
| BAL030 | *∆ywnE ∆ywjE ∆ywiE::kan ∆pssA::erm* | 25.64 ± 2.56 | This work |
| BAL031 | *∆pssA::erm* | 85.10 ± 12.16 | This work |
| BAL032 | *∆ltaSA::erm* | 113.8 ± 27.24 | This work |
| BAL033 | *∆ugtP::erm* | 74.04 ± 22.12 | This work |
| BAL034 | *∆mprF::erm* | 119.2± 47.44 | This work |
| BAL035 | *∆floA::erm* | 35.9 ±12.18 | This work |
| BAL036 | *∆floT::erm* | 103.8±11.54 | This work |
| BAL037 | *∆fisB::tet ∆ywnE ∆ywjE ywiE::kan ycgO::PspoIID-RBSfisB(5n)-mYFP -fisB (erm)* | n.d | This work |
| BDR2061 | *amyE::PxylA-gfp-mbl (spec), mblΩpMUTIN4 (erm) trpC2* | n.d | ^12^ |
| BMB014 | *amyE::PxylA-mciZ(cat), ycgO::Pspank-gfp-fisB (spec)* | n.d | This work |
| BMB031 | *ycgO::PfisB-RBSfisB-Myc-fisB(erm)* | 101 ± 7 | This work |
| BMB032 | *ycgO::PfisB-RBSfisB-Myc-fisB(G6C) (erm)* | 68 ± 19 | This work |
| BMB033 | *ycgO::PfisB-RBSfisB-Myc-fisB(L137C) (erm)* | 53 ± 3 | This work |
| BMB034 | *ycgO::PfisB-RBSfisB-Myc-fisB(A245C) (erm)* | 83 ± 8 | This work |
| BS168 | *Wild-type strain trpC2* | n.d | BGSC^b^ |
| 1A1246 | *amyE::(Pveg(+1/+8)_R1-18_sfGFP_spec) trpC2* | n.d | BGSC |
| 1A1243 | *amyE::(Pveg(+1/+8)_R1-15_sfGFP_spec) trpC2* | n.d | BGSC |
| 1A1227 | *amyE::(Pveg(+1/+8)_R0-16_sfGFP_spec) trpC2* | n.d | BGSC |
| 1A1241 | *amyE::(Pveg(+1/+8)_R1-13_sfGFP_spec) trpC2* | n.d | BGSC |
| 1A1239 | *amyE::(Pveg(+1/+8)_R1-11_sfGFP_spec) trpC2* | n.d | BGSC |
| 1A1237 | *amyE::(Pveg(+1/+8)_R1-9_sfGFP_spec) trpC2* | n.d | BGSC |
| 1A1220 | *amyE::(Pveg(+1/+8)_R0-9_sfGFP_spec) trpC2* | n.d | BGSC |
| SG13 | *amyE::(Pveg_R0_sfGFP_spec)* | n.d | BGSC |
| BAL038 | *amyE::(Pveg_R0_mEGFP_spec)* | n.d | This work |

**a:** Sporulation efficiency (% of WT spores at 24h after the onset of sporulation) for each indicated strain. Results are shown as means ± SD for four replicates per condition.

**b:** Bacillus Genetic Stock Center (www.bgsc.org)

**Table 3. Plasmids used in this study.**

| **Plasmid** | **Genotype** | **E.Coli** | **Source** |
| --- | --- | --- | --- |
| pAM002 | *ycgO::PfisB-mGFP -fisB (cat)* | amp | ^25^ |
| pVS001 | *ycgO::PfisB-mYFP A206K -fisB (cat)* | amp | This work |
| pAL001 | *ycgO::PfisB-mEGFP-fisB B.subtilis.(cat)* | amp | This work |
| pAL002 | *ycgO::PspoIID-RBSfisB(5n)-mEGFP-fisB (erm)* | amp | This work |
| pAL003 | *ycgO::PfisB-mEGFP-fisB Clostridium Perfringens sp.(cat)* | amp | This work |
| pAL004 | *ycgO::PspoIID-RBSfisB(5n)-mYFP A206K-fisB (erm)* | amp | This work |
| pAL005 | *ycgO::PspoIID-RBSfisB(5n)-mYFP A206K-fisB K168DK170D (erm)* | amp | This work |
| pAL006 | *ycgO::PspoIID-RBSfisB(5n)-mYFP A206K-fisB G175A I176S I194TI195S (erm)* | amp | This work |
| pKM110 | *his6-fisB^ECD^ WT* | amp | ^25^ |
| pAL007 | *his6-fisB^ECD^ WT* G123C | amp | This work |
| pDT390 | *his6-fisB^ECD^ G175A I176S I194T I195S* | amp | ^25^ |
| pAL008 | *his6-fisB^ECD^ G175A I176S I194T I195S G13C* | amp | This work |
| pAL009 | *his6-fisB^ECD^* K168D K170D | amp | This work |
| pAL010 | *his6-fisB^ECD^* K168D K170D G123C | amp | This work |
| pAL011 | *ycgO::PspoIID-RBSfisB(5n)-mYFP A206K-fisB ∆80-96 (erm)* | amp | This work |
| pAL012 | *ycgO::PspoIID-RBSfisB(5n)-mYFP A206K-fisB ∆122-132 (erm)* | amp | This work |
| pAL013 | *ycgO::PspoIID-RBSfisB(5n)-mYFP A206K-fisB ∆137-154 (erm)* | amp | This work |
| pAL014 | *ycgO::PspoIID-RBSfisB(5n)-mYFP A206K-fisB ∆167-182 (erm)* | amp | This work |
| pAL015 | *ycgO::PspoIID-RBSfisB(5n)-mYFP A206K-fisB ∆210-220 (erm)* | amp | This work |
| pAL016 | *ycgO::PspoIID-RBSfisB(5n)-mYFP A206K-fisB ∆132-222 (erm)* | amp | This work |
| pAL017 | *ycgO::PspoIID-RBSfisB(5n)-mYFP A206K-fisB L90T (erm)* | amp | This work |
| pAL018 | *ycgO::PspoIID-RBSfisB(5n)-mYFP A206K-fisB L137SG138A (erm)* | amp | This work |
| pAL019 | *ycgO::PspoIID-RBSfisB(5n)-mYFP A206K-fisB L145TL146S (erm)* | amp | This work |
| pAL020 | *ycgO::PspoIID-RBSfisB(5n)-mYFP A206K-fisB G150A (erm)* | amp | This work |
| pAL021 | *ycgO::PspoIID-RBSfisB(5n)-mYFP A206K-fisB V215SG217A (erm)* | amp | This work |
| pAL022 | *ycgO::PspoIID-RBSfisB(5n)-mYFP A206K-fisB V219T (erm)* | amp | This work |
| pAL023 | *ycgO::PspoIID-RBSfisB(5n)-mYFP A206K-fisB G175AI176S (erm)* | amp | This work |
| pAL024 | *ycgO::PspoIID-RBSfisB(5n)-mYFP A206K-fisB I194TI195S (erm)* | amp | This work |
| pAL025 | *ycgO::PspoIID-RBSfisB(5n)-mYFP A206K-fisB* R56E *(erm)* | amp | This work |
| pAL026 | *ycgO::PspoIID-RBSfisB(5n)-mYFP A206K-fisB E67RD68K (erm)* | amp | This work |
| pAL027 | *ycgO::PspoIID-RBSfisB(5n)-mYFP A206K-fisB K106D K109D (erm)* | amp | This work |
| pAL028 | *ycgO::PspoIID-RBSfisB(5n)-mYFP A206K-fisB K116D (erm)* | amp | This work |
| pAL029 | *ycgO::PspoIID-RBSfisB(5n)-mYFP A206K-fisB E119R (erm)* | amp | This work |
| pAL030 | *ycgO::PspoIID-RBSfisB(5n)-mYFP A206K-fisB R156E (erm)* | amp | This work |
| pAL031 | *ycgO::PspoIID-RBSfisB(5n)-mYFP A206K-fisB K170D K172DE (erm)* | amp | This work |
| pDR244 | PPA-cre-ori(ts) spec | amp | BGSC |
| pMB062 | *ycgO::PfisB-RBSfisB-Myc-fisB(erm)* | amp | This work |
| pMB064 | *ycgO::PfisB-RBSfisB-Myc-fisB(G6C) (erm)* | amp | This work |
| pMB065 | *ycgO::PfisB-RBSfisB-Myc-fisB(L137C) (erm)* | amp | This work |
| pMB066 | *ycgO::PfisB-RBSfisB-Myc-fisB(A245C) (erm)* | amp | This work |
| pVS002 | his6-SUMO-mYFP | kan | This work |
| pECE321 | Pveg_R0_sfGFP | amp | BGSC |
| pECE321_mEGFP | Pveg_R0_mEGFP | amp | This work |

**REFERENCES**

1. Guiziou S*, et al.* A part toolbox to tune genetic expression in Bacillus subtilis. *Nucleic Acids Res* **44**, 7495-7508 (2016).

2. Ojkic N, López-Garrido J, Pogliano K, Endres RG. Cell-wall remodeling drives engulfment during Bacillus subtilis sporulation. *Elife* **5**, (2016).

3. Carballido-Lopez R, Formstone A, Li Y, Ehrlich SD, Noirot P, Errington J. Actin homolog MreBH governs cell morphogenesis by localization of the cell wall hydrolase LytE. *Dev Cell* **11**, 399-409 (2006).

4. Carballido-Lopez R, Formstone A. Shape determination in Bacillus subtilis. *Curr Opin Microbiol* **10**, 611-616 (2007).

5. Strahl H, Hamoen LW. Membrane potential is important for bacterial cell division. *Proc Natl Acad Sci U S A* **107**, 12281-12286 (2010).

6. Meyer P, Gutierrez J, Pogliano K, Dworkin J. Cell wall synthesis is necessary for membrane dynamics during sporulation of Bacillus subtilis. *Mol Microbiol* **76**, 956-970 (2010).

7. Shaevitz JW, Gitai Z. The structure and function of bacterial actin homologs. *Cold Spring Harb Perspect Biol* **2**, a000364 (2010).

8. Garner EC, Bernard R, Wang W, Zhuang X, Rudner DZ, Mitchison T. Coupled, circumferential motions of the cell wall synthesis machinery and MreB filaments in B. subtilis. *Science* **333**, 222-225 (2011).

9. Schirner K*, et al.* Lipid-linked cell wall precursors regulate membrane association of bacterial actin MreB. *Nat Chem Biol* **11**, 38-45 (2015).

10. Silver LL. Fosfomycin: Mechanism and Resistance. *Cold Spring Harb Perspect Med* **7**, (2017).

11. Axelrod D. Total internal reflection fluorescence microscopy in cell biology. *Traffic* **2**, 764-774 (2001).

12. Carballido-López R, Errington J. The bacterial cytoskeleton: in vivo dynamics of the actin-like protein Mbl of Bacillus subtilis. *Dev Cell* **4**, 19-28 (2003).

13. Huet S, Karatekin E, Tran VS, Fanget I, Cribier S, Henry JP. Analysis of transient behavior in complex trajectories: application to secretory vesicle dynamics. *Biophys J* **91**, 3542-3559 (2006).

14. El-Gebali S*, et al.* The Pfam protein families database in 2019. *Nucleic Acids Res* **47**, D427-D432 (2019).

15. Bogdanov M, Heacock PN, Dowhan W. Study of polytopic membrane protein topological organization as a function of membrane lipid composition. *Methods Mol Biol* **619**, 79-101 (2010).

16. Ovchinnikov S*, et al.* Protein structure determination using metagenome sequence data. *Science* **355**, 294-298 (2017).

17. Sharp MD, Pogliano K. An in vivo membrane fusion assay implicates SpoIIIE in the final stages of engulfment during Bacillus subtilis sporulation. *Proc Natl Acad Sci U S A* **96**, 14553-14558 (1999).

18. Smith MB, Li H, Shen T, Huang X, Yusuf E, Vavylonis D. Segmentation and tracking of cytoskeletal filaments using open active contours. *Cytoskeleton (Hoboken)* **67**, 693-705 (2010).

19. Smith MB, Karatekin E, Gohlke A, Mizuno H, Watanabe N, Vavylonis D. Interactive, computer-assisted tracking of speckle trajectories in fluorescence microscopy: application to actin polymerization and membrane fusion. *Biophys J* **101**, 1794-1804 (2011).

20. Landau M*, et al.* ConSurf 2005: the projection of evolutionary conservation scores of residues on protein structures. *Nucleic Acids Res* **33**, W299-302 (2005).

21. Dobson L, Reményi I, Tusnády GE. CCTOP: a Consensus Constrained TOPology prediction web server. *Nucleic Acids Res* **43**, W408-412 (2015).

22. Sievers F*, et al.* Fast, scalable generation of high-quality protein multiple sequence alignments using Clustal Omega. *Mol Syst Biol* **7**, (2011).

23. Youngman PJ, Perkins JB, Losick R. Genetic transposition and insertional mutagenesis in Bacillus subtilis with Streptococcus faecalis transposon Tn917. *Proc Natl Acad Sci U S A* **80**, 2305-2309 (1983).

24. Eichenberger P*, et al.* The sigmaE regulon and the identification of additional sporulation genes in Bacillus subtilis. *J Mol Biol* **327**, 945-972 (2003).

25. Doan T*, et al.* FisB mediates membrane fission during sporulation in Bacillus subtilis. *Genes Dev* **27**, 322-334 (2013).
